# Phosphorylated MED1 links transcription recycling and cancer growth

**DOI:** 10.1093/nar/gkac246

**Published:** 2022-04-08

**Authors:** Zhong Chen, Zhenqing Ye, Raymond E Soccio, Tomoyoshi Nakadai, William Hankey, Yue Zhao, Furong Huang, Fuwen Yuan, Hongyan Wang, Zhifen Cui, Benjamin Sunkel, Dayong Wu, Richard K Dzeng, Jennifer M Thomas-Ahner, Tim H M Huang, Steven K Clinton, Jiaoti Huang, Mitchell A Lazar, Victor X Jin, Robert G Roeder, Qianben Wang

**Affiliations:** Department of Pathology and Duke Cancer Institute, Duke University School of Medicine, Durham, NC 27710, USA; Department of Molecular Medicine, University of Texas Health Science Center at San Antonio, San Antonio, TX 78229, USA; Division of Endocrinology, Diabetes, and Metabolism, Department of Medicine, Department of Genetics, and the Institute for Diabetes, Obesity, and Metabolism, Perelman School of Medicine at the University of Pennsylvania, Philadelphia, PA 19104, USA; Laboratory of Biochemistry and Molecular Biology, The Rockefeller University, New York, NY 10065, USA; Department of Pathology and Duke Cancer Institute, Duke University School of Medicine, Durham, NC 27710, USA; Department of Pathology and Duke Cancer Institute, Duke University School of Medicine, Durham, NC 27710, USA; Department of Pathology, College of Basic Medical Sciences and First Affiliated Hospital, China Medical University, Shenyang 110122, China; Department of Pathology and Duke Cancer Institute, Duke University School of Medicine, Durham, NC 27710, USA; Department of Pathology and Duke Cancer Institute, Duke University School of Medicine, Durham, NC 27710, USA; Department of Pathology and Duke Cancer Institute, Duke University School of Medicine, Durham, NC 27710, USA; Department of Pathology and Duke Cancer Institute, Duke University School of Medicine, Durham, NC 27710, USA; Department of Cancer Biology and Genetics, The Ohio State University College of Medicine, Columbus, OH 43210, USA; Department of Cancer Biology and Genetics, The Ohio State University College of Medicine, Columbus, OH 43210, USA; Division of Endocrinology, Diabetes, and Metabolism, Department of Medicine, Department of Genetics, and the Institute for Diabetes, Obesity, and Metabolism, Perelman School of Medicine at the University of Pennsylvania, Philadelphia, PA 19104, USA; Division of Medical Oncology, The Ohio State University Comprehensive Cancer Center, Columbus, OH 43210, USA; Department of Molecular Medicine, University of Texas Health Science Center at San Antonio, San Antonio, TX 78229, USA; Division of Medical Oncology, The Ohio State University Comprehensive Cancer Center, Columbus, OH 43210, USA; Department of Pathology and Duke Cancer Institute, Duke University School of Medicine, Durham, NC 27710, USA; Division of Endocrinology, Diabetes, and Metabolism, Department of Medicine, Department of Genetics, and the Institute for Diabetes, Obesity, and Metabolism, Perelman School of Medicine at the University of Pennsylvania, Philadelphia, PA 19104, USA; Department of Molecular Medicine, University of Texas Health Science Center at San Antonio, San Antonio, TX 78229, USA; Laboratory of Biochemistry and Molecular Biology, The Rockefeller University, New York, NY 10065, USA; Department of Pathology and Duke Cancer Institute, Duke University School of Medicine, Durham, NC 27710, USA

## Abstract

Mediator activates RNA polymerase II (Pol II) function during transcription, but it remains unclear whether Mediator is able to travel with Pol II and regulate Pol II transcription beyond the initiation and early elongation steps. By using *in vitro* and *in vivo* transcription recycling assays, we find that human Mediator 1 (MED1), when phosphorylated at the mammal-specific threonine 1032 by cyclin-dependent kinase 9 (CDK9), dynamically moves along with Pol II throughout the transcribed genes to drive Pol II recycling after the initial round of transcription. Mechanistically, MED31 mediates the recycling of phosphorylated MED1 and Pol II, enhancing mRNA output during the transcription recycling process. Importantly, MED1 phosphorylation increases during prostate cancer progression to the lethal phase, and pharmacological inhibition of CDK9 decreases prostate tumor growth by decreasing MED1 phosphorylation and Pol II recycling. Our results reveal a novel role of MED1 in Pol II transcription and identify phosphorylated MED1 as a targetable driver of dysregulated Pol II recycling in cancer.

## INTRODUCTION

The Mediator complex functions to bridge transcription factors with the preinitiation complex (PIC) and regulates the assembly and function of the PIC (1). Evidence has emerged that Mediator also regulates early transcription elongation (2,3). For example, Mediator overcomes the block of TFIIF–RNA polymerase II (Pol II) interactions by pausing factor Gdown1, thereby facilitating elongation (4,5). In addition, MED26 and CDK8–Mediator stimulate transcription elongation by recruiting P-TEFb and the super elongation complex (SEC) to promote the release of paused Pol II (6–8). However, it has remained unclear whether Mediator subunit is able to enter gene bodies, move along with Pol II and regulate Pol II transcription beyond the initiation and early elongation steps.

Cancer cells are addicted to aberrant Pol II transcription (9). For example, overexpression of Mediator subunit CDK8 enhances β-catenin-driven transcription initiation to promote colon cancer growth (10). In a second example, fusion of SEC subunits (e.g. ELL, AFF1 and AFF4) with the N-terminal region of the chromatin regulator MLL stimulates transcription elongation of MLL target genes (e.g. *HOXA9* and *HOXA10*), which leads to aggressive acute leukemia (11,12). Moreover, overexpression of the transcription factor c-Myc promotes the release of poised Pol II and enhances transcription elongation of most actively transcribed genes, which facilitates growth of diverse types of cancer cells (13,14). While these studies indicate that uncontrolled transcriptional initiation and elongation have oncogenic roles, it is currently unknown whether other Pol II transcription processes contribute to cancer growth. Interestingly, we have recently found that enhanced Pol II recycling (i.e. Ser2 phosphorylated Pol II [Pol II (Ser2)] dynamically recycling after the initial transcription cycle), a fundamental transcriptional process that significantly affects mRNA output, is associated with prostate tumorigenesis (15). However, the causal link between aberrant Pol II recycling and cancer growth has not been established.

Here, using our newly developed transcription recycling assays (15), we report the finding that human Mediator is involved in Pol II recycling. We further show that CDK9-phosphorylated MED1 (pMED1) travels with Pol II in transcription cycles, drives Pol II recycling and enhances mRNA output. We have also demonstrated that MED31 mediates interactions between pMED1 and Pol II and augments transcription recycling. Importantly, MED1 phosphorylation is significantly increased during prostate cancer progression to the lethal castration-resistant phase [castration-resistant prostate cancer (CRPC)]. Targeting MED1 phosphorylation by a highly specific CDK9 inhibitor decreases tumor growth through inhibition of Pol II recycling. Our study identifies pMED1 as a targetable molecular linker between Pol II recycling and tumor growth.

## MATERIALS AND METHODS

### Mammalian cell lines

The human male LNCaP-abl cell line has been used in previous studies as a model for CRPC (16,17), and was authenticated as described previously (18). 22Rv1 and C4-2 CRPC cell models (19,20) were obtained from American Type Culture Collection (ATCC). Cells were cultured in RPMI 1640 (Invitrogen) supplemented with 10% charcoal-stripped fetal bovine serum (FBS) or 10% FBS (Omega Scientific) and 1 mM l-glutamine. The human female Flp-In™ T-REx™-293 cell line was purchased from Life Technologies (R78007) and cultured in Dulbecco’s modified Eagle medium (DMEM), high glucose with 10% FBS and 2 mM l-glutamine (Invitrogen). Mouse male 3T3-L1 cells (ATCC) were grown in DMEM (Invitrogen) with 10% FBS (Tissue Culture Biologics), 100 U/ml penicillin and 100 mg/ml streptomycin (Invitrogen). Two days post-confluence, differentiation medium [growth medium with 1 mM dexamethasone, 10 mg/ml human insulin and 0.5 mM 3-isobutyl-1-methylxanthine (Sigma)] was added. To generate adipocytes, cells were grown in differentiation medium for 3 days, followed by growth medium with 10 mg/ml insulin for 3 days, followed by growth medium only. After 8 days, >90% of cells had accumulated lipid droplets by phase contrast microscopy. All cultures were incubated at 37°C in 5% CO_2_. All cell lines have been authenticated prior to commencing this study by short tandem repeat profiling and karyotyping. All cell lines were routinely tested to ensure that they were free of mycoplasma contamination (Venor™ GeM Mycoplasma Detection Kit, Sigma-Aldrich).

### Human tissues

The prostate cancer tissue microarrays including 141 normal prostate specimens, 74 androgen-dependent prostate cancer (ADPC) specimens and 19 CRPC specimens were generated under the supervision of Dr Jiaoti Huang. All experimental procedures were approved by Duke University Institutional Review Boards. The prostate tissues used for pMED1 chromatin immunoprecipitation sequencing (ChIP-seq) were obtained from The Ohio State University (OSU) Wexner Medical Center under the approval of the OSU Institutional Review Board. Radical prostatectomy specimens were snap-frozen in liquid nitrogen immediately after surgical excision. The frozen tissue samples of liver and lung were both purchased from Cureline, Inc. Small pieces of tissue prepared by a certified medical pathologist were snap-frozen in liquid nitrogen within 4–12 h postmortem in autopsy cases. All tissues were stored at −80°C until they were used.

### Animal models

Male, 5-week-old, athymic Balb/c nude mice were obtained from Charles River Laboratories and acclimated for 1 week in a pathogen-free enclosure before the start of the study. Eleven mice per experimental arm were randomly assigned and used for xenograft studies. All experiments were conducted in accordance with the guidelines of the Association for Assessment and Accreditation of Laboratory Animal Care International and the protocol approved by the Duke University Medical Center Institutional Animal Care and Use Committee.

### *In vitro* transcription recycling assay

Nuclear protein extraction from 293 or LNCaP-abl cells, linear DNA template amplification and immobilization, *in vitro* transcription and recycling, and RNA quantification by qPCR were all performed as we have previously described (15) (Supplementary Materials and Methods).

### Two-dimensional difference gel electrophoresis

Two-dimensional difference gel electrophoresis (2D DIGE) was performed as we have described previously (15) (Supplementary Materials and Methods).

### Nano-liquid chromatography coupled with tandem mass spectrometry

Nano-LC–MS/MS (nano-liquid chromatography coupled with tandem mass spectrometry) analysis was performed as we have described previously (15) (Supplementary Materials and Methods).

### ChIP and ChIP-seq

Mouse 3T3-L1 adipocytes were treated with 1 μM rosiglitazone or vehicle [dimethyl sulfoxide (DMSO)] for 1 h. Human LNCaP-abl cells were either treated with flavopiridol (FP) (1 μM or 300 nM) or vehicle (DMSO) over a time course. ChIP-seq was performed as previously described (21) (Supplementary Materials and Methods).

### RNA-seq

RNA-seq analysis was performed as previously described (22) (Supplementary Materials and Methods).

### RNA interference

For transient knockdown of MED31, ON-TARGETplus *MED31* siRNA (L-027282-00-0020) and ON-TARGETplus Non-targeting Pool (D-001810-10-50) were purchased from Dharmacon. A total of 5 × 10^6^ LNCaP-abl cells were plated in 15-cm dishes and then transfected with Lipofectamine^®^ RNAiMAX Reagent (Invitrogen) according to the protocol provided by the manufacturer. After 3 days, nuclear proteins were extracted using the NE-PER Nuclear and Cytoplasmic Extraction Kit (Thermo Scientific) according to manufacturer’s instructions.

### Mammalian *in vitro* translation assay

Human full-length MED1 and RPB1 were expressed using the 1-Step CHO High-Yield IVT Kit (Thermo Scientific). Briefly, after reconstitution of the lyophilized CHO lysate, the *in vitro* translation reaction was set up by incubating CHO lysate with accessory proteins for 10 min at room temperature before adding reaction buffer and plasmids. After centrifuging the reaction mix at 10 000 × *g* for 2 min, the supernatant was transferred into the empty dialysis device and incubated for 16 h at 30°C in a shaker incubator (350 rpm). At the end of incubation, proteins were collected after centrifuging at 10 000 × *g* for 2 min and stored at −80°C.

### GST pull-down assay

Sequences of human MED4, MED9 and MED31 were cloned in frame into SgfI–PmeI sites of the Promega Flexi vector. All constructs were verified by DNA sequencing. The resulting plasmids were then transfected into KRX competent cells (Cat #L3002, Promega) and expression of GST–MED4, GST–MED9 and GST–MED31 protein fusions was induced by 0.1% rhamnose overnight according to the manufacturer’s protocol (Promega). The bacteria were lysed with lysis buffer and the GST fusion proteins were bound to magneGST particles. Five microliters of *in vitro* translated MED1 or RPB1 per reaction was incubated with the immobilized GST fusion proteins (5 μl of GST fusion protein immobilized magneGST particles per reaction) in a total volume of 200 μl of magneGST binding/washing buffer at 4°C with rotation. After 1 h of incubation, the beads were washed five times with 250 μl ice-cold binding/washing buffer and proteins remaining bound to the immobilized GST fusion proteins were eluted with 20 μl of 2× SDS loading buffer and the eluted proteins were resolved by SDS-PAGE. MED1 or RPB1 western blot was then performed.

### *In vitro* CDK9 kinase assay

*In vitro* kinase assays were performed using 25 μl of kinase reaction. Five nanograms of CDK9/CyclinK (Invitrogen) was incubated with 20 ng baculovirus recombinant full-length MED1 in 10 μM ATP, 40 mM Tris (pH 7.5), 20 mM MgCl_2_, 0.1 mg/ml BSA and 50 μM DTT. DMSO or 1 μM FP was added prior to starting the assay. After 1 h of incubation at 25°C, reactions were stopped by adding an equal volume of 2× SDS sample buffer and heating to 95°C for 5 min.

### Co-immunoprecipitation

Nuclear extracts were prepared using the NE-PER Nuclear and Cytoplasmic Extraction Kit (Thermo Scientific). To co-immunoprecipitate pMED1 with CDK9 from LNCaP-abl nuclear extracts, the lysates were diluted with 25 mM Tris (pH 7.4), 0.15 M NaCl, 1 mM EDTA, 1% NP-40 and 5% glycerol. Antibodies conjugated with protein A beads were added and incubated for 2 h at 4°C. The beads were then washed three times with 50 mM Tris (pH 8.0), 150 mM NaCl and 0.2% NP-40. Bound proteins were separated on SDS-PAGE gels and immunoblotted with antibodies against CDK9 and pMED1. To test pMED1 binding to PAF1, SUPT5H or Pol II (Ser2), nuclear extracts were incubated with pMED1 antibody overnight at 4°C. The next day, protein A beads were added and incubated for 2 h, followed by washing six times with buffer (25 mM Tris, pH 8.0, 150 mM NaCl, 1 mM EDTA and 1% NP-40).

### Western blotting

Protein samples were boiled for 5 min and then resolved in 4–15% or 7.5% mini-PROTEAN TGX Gels (Bio-Rad). Proteins were transferred to PVDF membranes using a semi-dry transfer cell or Trans-Blot cell (Bio-Rad). Membranes were blocked with Azure Chemi Blot Blocking Buffer (Azure) at room temperature for 1 h, and then incubated with the appropriate primary antibodies at 4°C overnight, followed by washing and incubation of secondary antibodies at room temperature for 1 h (see Supplementary Data for antibody information). Immunoblot signal was developed with chemiluminescent substrate for quantitative chemiluminescent Westerns (Azure) and captured using the C-DiGit Chemiluminescent Western Blot Scanner (Li-Cor) or Azure Western Blot Imaging System (Azure). Western blots were performed at least twice.

### Cell proliferation assays

A total of 2 × 10^5^ LNCaP-abl or 22Rv1 cells were plated overnight and transfected with wild-type (WT) MED1, MED1 (T1032A) or MED1 (T1032D) plasmids using Lipofectamine 3000 Transfection Reagent (Invitrogen) according to the manufacturer’s protocol with a DNA to Lipofectamine ratio of 1:3 (w/v). The P3000 enhancer reagent (1:2, DNA:reagent, w/v) was used along with the Lipofectamine 3000 Transfection Reagent for all transfections. Cell number in each well was determined by cell counting using 0.1% trypan blue on days as indicated. Each data point represents mean ± SD of at least two replicates.

### Immunohistochemistry analysis

Tumor microarrays containing normal prostate (141 samples), ADPC (74 samples) or CRPC (19 samples) were constructed in the Duke University Department of Pathology under the supervision of Dr Jiaoti Huang. Immunohistochemical staining of these samples was performed in the Duke University Department of Pathology with a phospho-specific pMED1 T1032 rabbit polyclonal antibody developed by our lab (23). Briefly, following deparaffinization, antigen retrieval was performed for 40 min using Reveal Decloaker solution (Biocare Medical), followed by cooling for 20 min. This was followed by application of Protein Block (Biocare Medical) for 15 min and Endogenous Peroxidase Quench (Biocare Medical) for 6 min. Primary antibody was applied for 60 min at a dilution of 1:250, while secondary antibody detection was performed as part of the MACH 4™ detection system (Biocare Medical). Counterstaining was performed with hematoxylin. Slides were digitally scanned at 20× magnification using a whole slide scanner (Leica) by the Duke Image Cytometry Lab. *H*-scores were assigned by Dr Yue Zhao and reviewed by Dr Jiaoti Huang of the Duke University Department of Pathology. These scores ranged from 0 to 300 and were calculated as the product of the intensity score for the epithelial region of maximum pMED1 staining intensity in each sample (assigned on a scale from 0 to 3) multiplied by the percentage of epithelial cells in that sample showing maximum staining intensity (0–100%).

### Mouse xenograft studies

Mouse xenograft studies were performed as described previously (24). LNCaP-abl cells (3 × 10^6^ cells/flank) were suspended with 50% Matrigel (Becton Dickinson, Franklin Lakes, NJ) and subcutaneously inoculated into the mouse flanks, monitored daily and tumor size quantified with calipers twice a week. Nineteen days after cell inoculation (tumors were grown to 60 mm^3^), treatments were initiated. Mice were randomly assigned into two cohorts with 11 mice per group. Atuveciclib (treated group) or vehicle solution (40% polyethylene glycol 400) (Sigma) was administered by oral gavage at the dose of 25 mg/kg once daily (25). The injection volume was 10 ml/kg. Tumor volume was calculated by using the standard formula: *V* = length × width^2^ × 0.5. Body weight was also monitored twice weekly. After 2 weeks, mice were euthanized and tumor tissues were weighed. Western blot analyses of pMED1, Pol II (Ser2) and histone H3 (control) were performed using nuclear extracts from pooled tissues.

### Statistical analysis

All quantification and statistical analyses were performed using SigmaPlot (13.0), Partek Genomics Suite (7.18) and Peaks Studio (10.0). Data are shown as mean ± SEM of biological duplicates or triplicates. Details of the statistical analysis for each experiment can be found in the relevant figure legends. All statistical analyses were calculated using two-tailed Student’s *t*-test unless otherwise mentioned.

## RESULTS

### pMED1 is involved in transcription recycling *in vitro*

**Figure 1.**
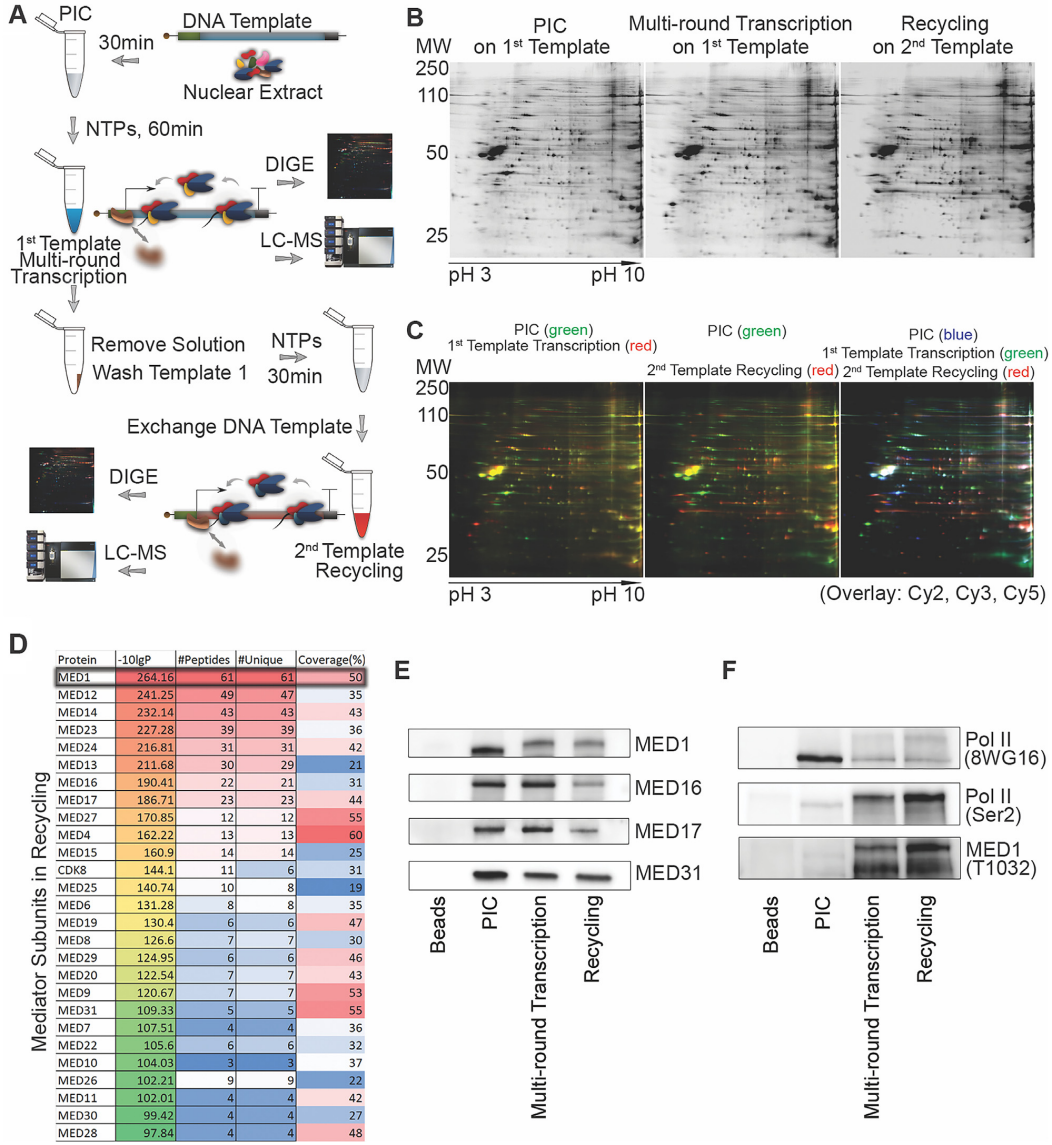
Mediator and pMED1 are involved in transcription recycling *in vitro*. **(A)** Schematic of the *in vitro* transcription recycling assay. **(B)** Representative two-dimensional gels showing protein profiles bound to templates as indicated. **(C)** DIGE comparison of template-bound proteins. **(D)** Template-bound proteins identified by LC–MS/MS in recycling. **(E)** Selected Mediator subunits bound to templates were validated by western blot. **(F)** Template-bound Pol II, phosphorylated Pol II and phosphorylated MED1 were examined by western blot.

To elucidate the process of transcription recycling at a mechanistic level, we recently developed an *in vitro* two-template transcription recycling system to isolate putative transcription recycling proteins (Figure 1A) (15). We separated proteins bound to the first template (PIC and multi-round transcription phases) or the second template (recycling phase) by DIGE (Figure 1B and C). Further LC–MS/MS analysis revealed that Mediator subunits were among the most enriched proteins on the second recycling template (Figure 1D, Supplementary Figure S1A–C and Supplementary Table S1). Among the Mediator subunits, the statistical significance of MED1 was the highest (Figure 1D and Supplementary Figure S1C). We next performed western blotting for selected Mediator subunits to confirm the proteomics findings and study dynamic changes of Mediator on the two templates. MED16 and MED17 remained on the first template from PIC formation to completion of multi-round transcription (Figure 1E), suggesting that these subunits may form a scaffold complex on the promoter (26). Interestingly, MED1 and MED31 partially dissociated from template during multi-round transcription and remained on the recycling template (Figure 1E and Supplementary Figure S1D), suggesting that at least some MED1 and MED31 molecules ran off or dropped off from the templates and participated in transcription recycling. Unexpectedly, we found that the molecular weight of MED1 was increased in multi-round transcription and recycling samples compared with PIC (Figure 1E), suggesting that post-translational modification of MED1 occurs during transcription. In view of the essential role of MED1 phosphorylation in promoting transcription (23,27), we next examined the levels of pMED1 on the templates. MED1 phosphorylation was markedly increased after the start of transcription and remained during recycling, paralleling the levels of Pol II (Ser2) (Figure 1F). These findings indicate that phosphorylation of MED1 and Pol II persists during multi-round transcription and that pMED1 and phosphorylated Pol II participate in transcription recycling *in vitro*.

### pMED1 binding to gene bodies of actively transcribed genes *in vivo*

**Figure 2.**
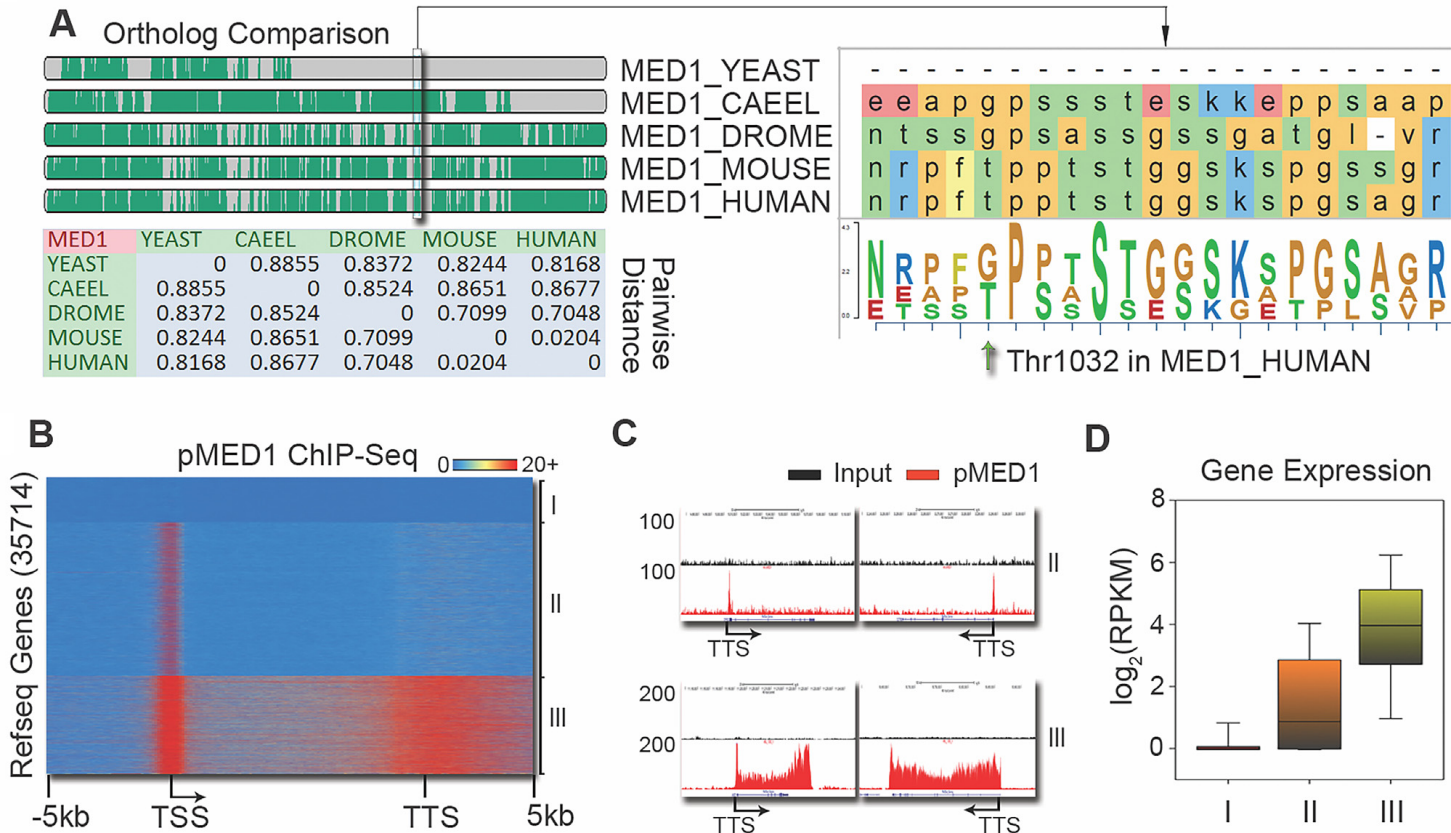
Genome-wide localization of pMED1. **(A)** Threonine phosphorylation is specifically gained in mammals. Ortholog comparison of MED1 protein sequences surrounding human T1032 (green arrow) was performed in yeast, *Caenorhabditis elegans*, *Drosophila*, mouse and human. **(B)** A heatmap illustrates the distribution of pMED1 ChIP-seq signals on scaled RefSeq genes in human LNCaP-abl cells. **(C)** UCSC Genome Browser views of pMED1 occupancy on representative class II/III genes in human LNCaP-abl cells. **(D)** Comparison of expression levels among the three classes of genes.

Having identified the involvement of pMED1 in transcription recycling *in vitro* (Figure 1), we next asked whether pMED1 binds to gene bodies on chromatin. While previous studies have found that yeast Mediator does not travel with Pol II into gene bodies (1,28), we found that human MED1, when phosphorylated at mammal-specific threonine (T)1032 (Figure 2A), can bind to the bodies of certain genes (e.g. *UBE2C*) in addition to binding to gene promoters (23). This potential evolutionary gain of function for MED1 prompted us to examine the genomic distribution of pMED1. We first performed pMED1 ChIP-seq in LNCaP-abl CRPC cells using a MED1 antibody that specifically recognizes MED1 phosphorylated at T1032 (23,29). Analysis of pMED1 localization over RefSeq annotated genes identified three classes of genes based on pMED1 distributions: class I genes lacked a detectable pMED1 signal, class II genes exhibited only promoter pMED1 occupancy and class III genes showed strong pMED1 binding from their 5′- to 3′-end regions (Figure 2B). Closer inspection of several examples of pMED1 binding to class II/III genes further confirmed this distinction (Figure 2C). We next investigated whether this distribution of pMED1 is limited to cultured cells by performing pMED1 ChIP-seq in human prostate, liver and lung tissues. Enrichment of pMED1 on gene bodies/3′-end regions was observed in all three types of tissues (Supplementary Figure S2A). Finally, analysis of pMED1 ChIP-seq in mouse 3T3-L1 adipocytes found that mouse genes can also be classified into the same three categories based on pMED1 distributions (Supplementary Figure S2B–D). These data reveal a distinct genomic distribution of pMED1 in mammalian cells and tissues. To ask whether pMED1 and unphosphorylated MED1 bind differentially over genes, we conducted ChIP-seq using a total MED1 antibody that primarily recognizes unphosphorylated MED (23) in LNCaP-abl cells. Total MED1 was recruited strongly to enhancers, consistent with previous reports (30,31) (Supplementary Figure S2E–G). Although we clearly observed total MED1 binding in gene bodies, the weak total MED1 signal was only weakly (though significantly) correlated with pMED1 signals from 5′- to 3′-end regions of class III genes (Supplementary Figure S2H and I). These results indicate that the genomic distribution of MED1 differs for its unphosphorylated and phosphorylated forms.

Because the distribution and density of pMED1 vary among genes, we postulated that pMED1 binding may reflect the status and levels of gene transcription. We addressed this in LNCaP-abl cells in three different ways. First, we performed ChIP-seq analysis of Ser5 and Ser2 phosphorylated Pol II, which mark transcriptional initiation and elongation, respectively (32,33). The levels of Pol II (Ser2) were high in gene bodies and further increased around the 3′-end regions of class III genes (Supplementary Figure S2J and K), suggesting that class III genes are undergoing active transcriptional elongation, 3′-end processing of RNA and transcription termination (34,35). The low level of Pol II (Ser2) and moderate level of Pol II (Ser5) on class II genes (Supplementary Figure S2J) suggest that this class is primed for active transcription. These data indicate that phosphorylated Pol II precisely coincides with pMED1 across transcribed genes. We next performed ChIP-seq of active histone marks reflecting transcriptional status (36,37). In accordance with Pol II distribution, class III genes showed the highest levels of H3K4me2, H3K4me3 and H3K27ac within promoters, as well as H3K36me3 and H3K79me2 within gene bodies (Supplementary Figure S2J and K). Further global correlation analysis demonstrated that pMED1 signal densities were highly correlated with the signal densities of both Ser2 phosphorylated Pol II and H3K36me3 (Supplementary Figure S2L). Finally, we performed RNA-seq analysis in LNCaP-abl cells. Class III genes, as indicated by high levels of pMED1, Pol II (Ser2) and active histone marks from 5′- to 3′-end regions, represent actively transcribed genes exhibiting the highest mRNA expression levels (Figure 2D). Further Gene Ontology pathway analysis revealed that the most enriched pathways among class III genes were metabolic and biosynthetic processes as well as transcriptional regulation, while cell signaling and developmental processes were identified as the most enriched pathways among class I and II genes (Supplementary Figure S2M). Together, our genomic localization data from Pol II and histone marks as well as functional gene expression data indicate that pMED1 occupancy throughout genes is a novel characteristic of highly transcribed genes.

### Intimate genomic interaction between pMED1 and Pol II (Ser2)

**Figure 3.**
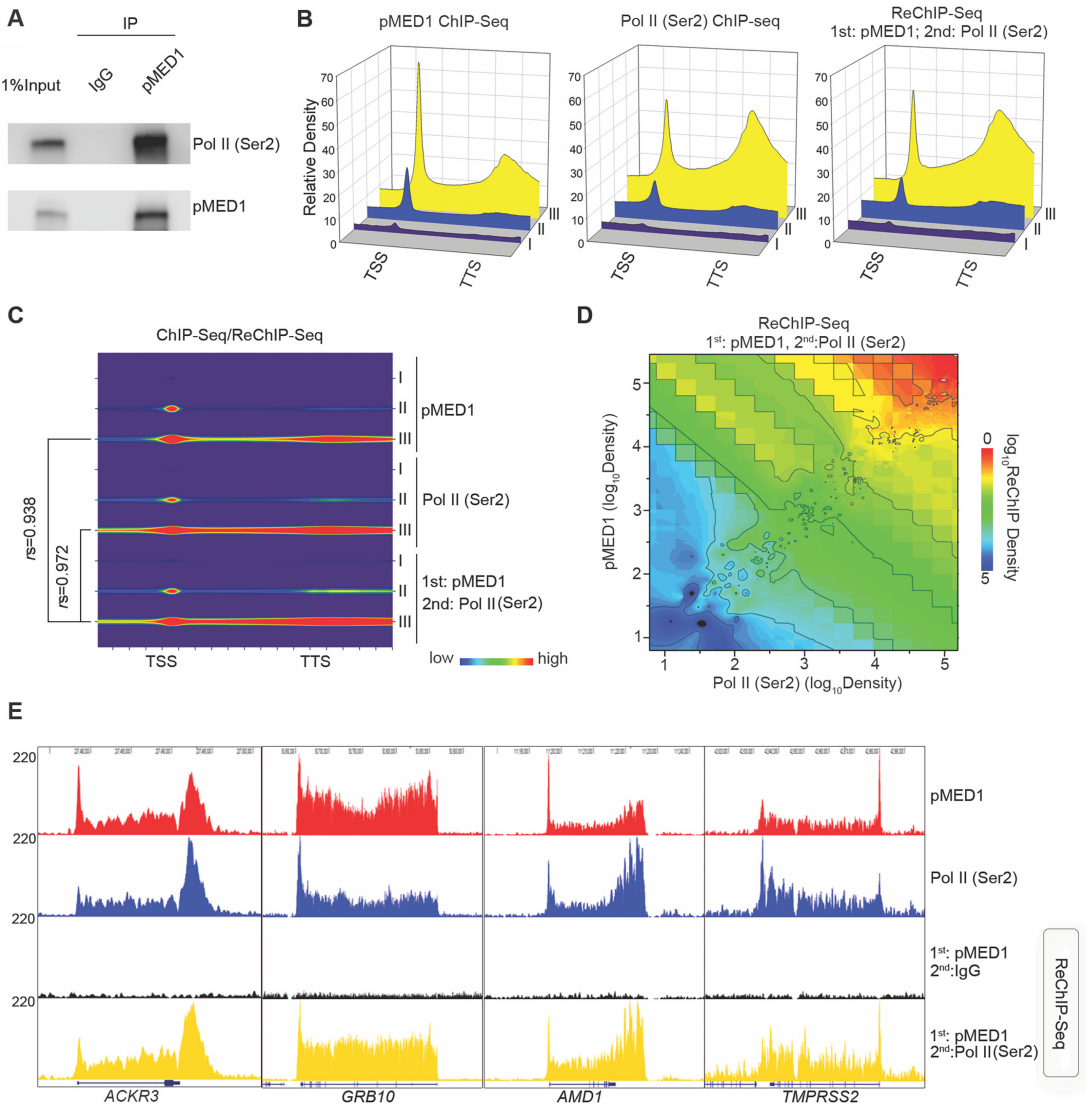
pMED1 interacts with Pol II (Ser 2) physically and genomically. **(A)** Nuclear extracts from LNCaP-abl cells were immunoprecipitated with the pMED1 antibody or IgG, and western blot analyses were performed with antibodies against Pol II (Ser2) or pMED1. **(B)** Comparisons of average signal densities of pMED1 ChIP-seq, Pol II (Ser2) ChIP-seq and pMED1/ Pol II (Ser2) Re-ChIP-seq on the three classes of genes. **(C)** Correlation analysis of pMED1/ Pol II (Ser2) Re-ChIP-seq signals with pMED1 or Pol II ChIP-seq signals throughout the three classes of genes. Normalized ChIP-seq read counts were calculated and plotted for all transcripts from TSS to TTS. For class II genes, *r*= 0.842 (pMED1 versus Re-ChIP) and *r*= 0.728 [Pol II (Ser2) versus Re-ChIP]. **(D)** Correlation of ChIP-seq signal density in class III genes based on pMED1, Pol II (Ser2) and pMED1/Pol II (Ser2) Re-ChIP-seq data. Normalized total ChIP-seq read counts of each gene were used to generate the matrix. 3D matrix was generated from Kriging correlation. **(E)** UCSC Genome Browser views of pMED1 ChIP-seq signals, Pol II ChIP-seq signals and pMED1 ChIP followed by Pol II (Ser2) Re-ChIP-seq signals at the *ACKR3*, *GRB10*, *AMD1* and *TMPRSS2* loci.

While pMED1 binds to gene bodies and coincides with Pol II (Ser 2) across transcribed genes (Figure 2 and Supplementary Figure S2), it is unknown whether pMED1 interacts with Pol II (Ser 2). Co-immunoprecipitation assays revealed that pMED1 was physically associated with Pol II (Ser2) (Figure 3A). To address whether pMED1 globally interacts with Pol II (Ser2) on chromatin, pMED1 ChIP followed by Pol II (Ser2) Re-ChIP-seq was performed. Integrative analysis of signal densities of pMED1 and Pol II (Ser2) ChIP-seq as well as Re-ChIP-seq not only confirmed that pMED1 precisely colocalized with Pol II (Ser2), but more importantly revealed that pMED1 strongly interacted with Pol II (Ser2) on the same DNA fragments *in vivo* (Figure 3B–E). Taken together, these findings indicate that pMED1 interacts with Pol II (Ser 2) physically and genomically.

### Dynamic traveling and recycling of pMED1 and Pol II (Ser2) *in vivo*

**Figure 4.**
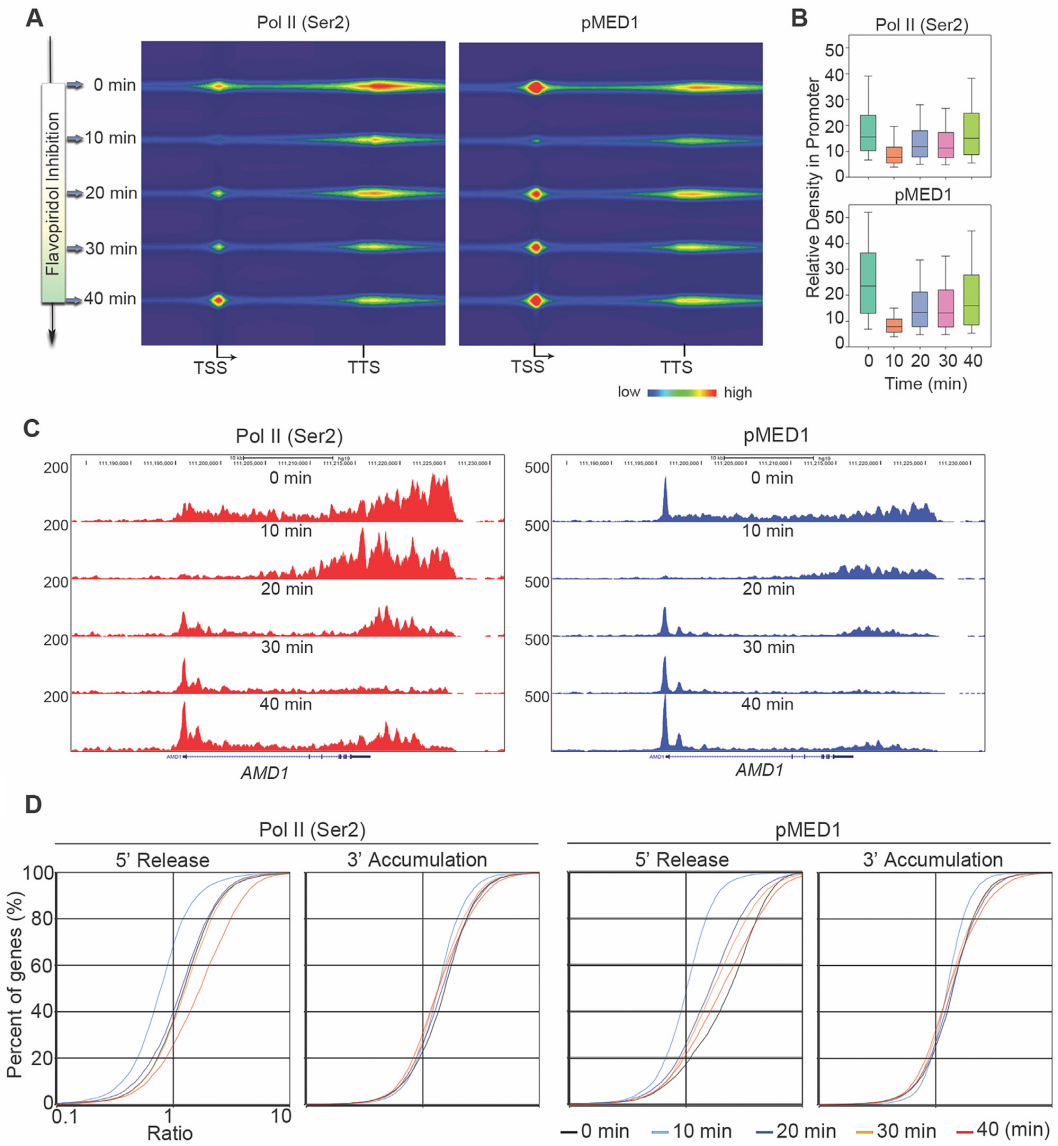
Dynamic analysis of pMED1 and Pol II (Ser 2) tracking on genes during FP inhibition. **(A)** Left: a schematic illustration of experimental setup. Right: heatmaps illustrate Pol II (Ser2) and pMED1 ChIP-seq signal changes in LNCaP-abl cells with timed FP inhibition. Normalized ChIP-seq read counts were calculated and plotted for all class III genes from TSS to TTS. **(B)** Box plots show Pol II (Ser2) and pMED1 ChIP-seq signal densities within −200 to 350 bp regions around the TSS during timed FP inhibition. **(C)** UCSC Genome Browser views of the binding of pMED1 and Pol II (Ser2) over *AMD* during FP inhibition. **(D)** 5′-Release ratios and 3′-accumulation ratios of Pol II (Ser2) and pMED1 were calculated for class III genes, sorted and plotted in LNCaP-abl cells with timed FP inhibition.

The intimate genomic interaction between pMED1 and Pol II (Ser2) motivated us to investigate whether pMED1 dynamically moves along with Pol II (Ser2). We determined whether pMED1 and Pol II (Ser2) are able to travel together by timed inhibition of Pol II escape into elongation (14,38). Pol II (Ser2) and pMED1 ChIP-seq were performed using the same population of cells treated for 0, 10, 20, 30 or 40 min with 300 nM FP, a ubiquitous inhibitor for productive elongation (38,39). After FP inhibition, the synchronous retreat of signals along gene bodies was observed between Pol II (Ser2) and pMED1 (Figure 4A–C), indicating that pMED1 travels along with Pol II (Ser2) during transcriptional elongation and termination. Surprisingly, we found that the densities of Pol II (Ser2) and pMED1 were increased synchronously and continuously in 5′-end regions after 20-min FP treatment (Figure 4A–D). As FP inhibits newly occurring Pol II phosphorylation at Ser2 but does not affect existing Pol II (Ser2) in the gene bodies (38), these data suggest that elongating Pol II rather than total Pol II recycles back to and accumulates in the 5′-end region following termination. Interestingly, pMED1 and Pol II (Ser2) patterns are cyclical and simultaneous (Figure 4A–D). These data further demonstrate that pMED1 dynamically travels with Pol II (Ser2) during transcription recycling *in vivo*.

### Mechanisms for pMED1 regulation of transcription recycling

**Figure 5.**
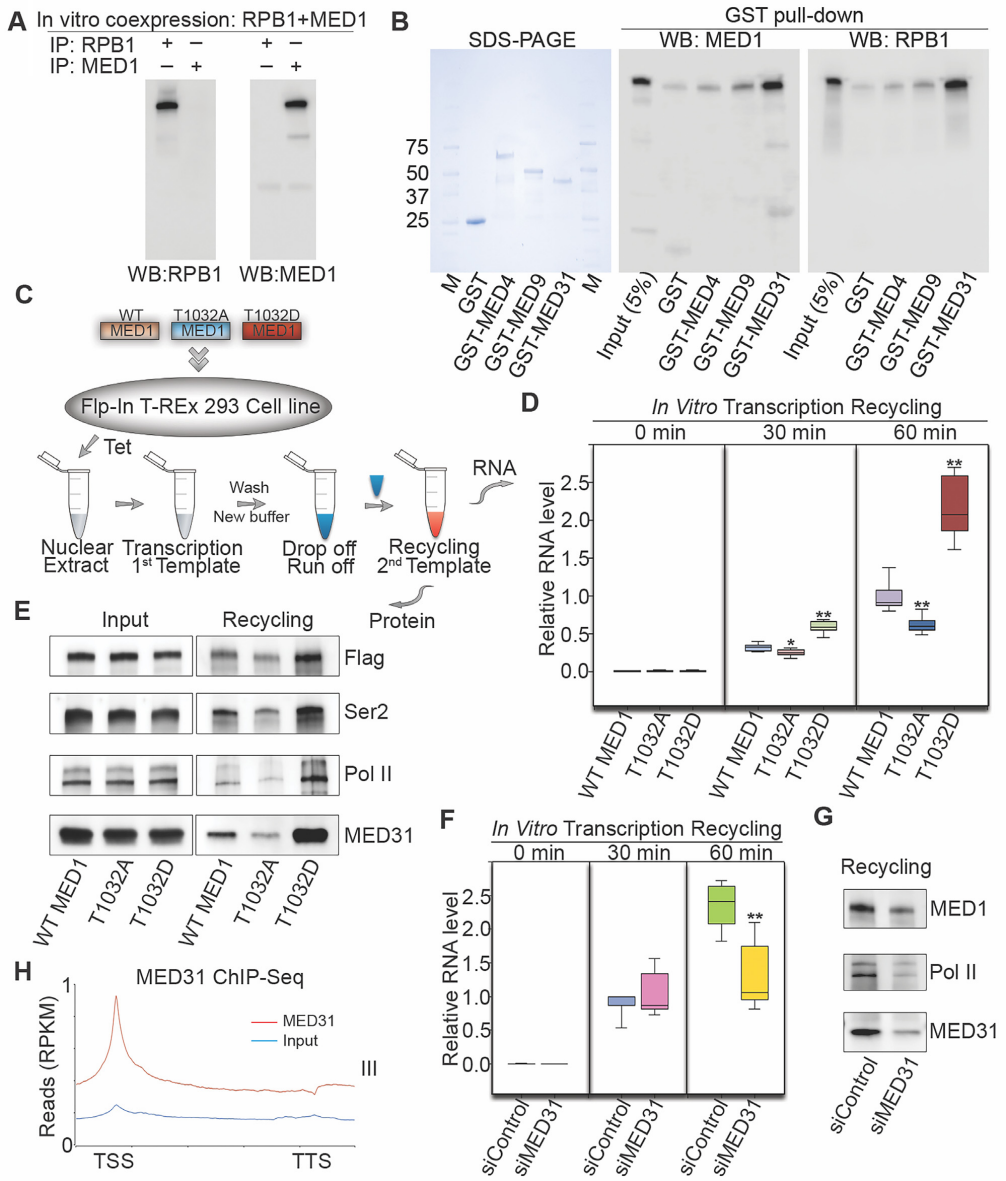
MED31 bridges pMED1 to Pol II in transcription recycling. **(A)** Full-length MED1 or RPB1 was expressed using an *in vitro* cell-free expression system. Purified proteins were incubated overnight and immunoprecipitated with RPB1 or MED1 antibodies, and analyzed by western blot. **(B)** GST pull-down assays were performed by incubating *in vitro* translated MED1 or RPB1 with the indicated GST fusion proteins. Western blots were performed using MED1 and RPB1 antibodies. **(C)** Schematic of transcription recycling assay. Flp-In 293 cell lines were generated stably expressing one single copy of WT or mutated FLAG-MED1. **(D)** RNA products from the second template reaction were analyzed by reverse transcription followed by qPCR quantification. Regions = 7 (*n* = 2), *P*-values were calculated using two-tailed Student’s *t*-test, *P < 0.01, **P < 0.001. **(E)** Proteins bound to the second template were assayed by western blots. **(F)** The transcription recycling assay was performed using nuclear extracts from siControl or siMED31-treated LNCaP-abl cells. RNA products from the second template reaction were analyzed by reverse transcription followed by qPCR quantification. Regions = 7 (*n* = 2), *P*-values were calculated using two-tailed Student’s *t*-test, **P < 0.001. **(G)** Proteins bound to the second template were assayed by western blotting. **(H)** Average MED31 ChIP-seq signal densities over the scaled human RefSeq genes of class III in LNCaP-abl cells.

In an effort to investigate the mechanistic role played by pMED1 in Pol II recycling, we first examined whether MED1 directly interacts with Pol II. *In vitro* co-immunoprecipitation of *in vitro* translated MED1 and the Pol II subunit RPB1 failed to reveal a direct interaction between these two proteins (Figure 5A), suggesting that MED1 may interact with Pol II via other Mediator subunits. Given that structural studies of *Schizosaccharomyces pombe* and *Saccharomyces cerevisiae* Mediator reveal that Med9, Med4 and Med1 form the plank domain in the Mediator middle module (40), and in view of the physical interaction between *S. cerevisiae* Med31, a knob domain subunit in the middle module, and Pol II Rpb1 (41), we next expressed MED4, MED9 and MED31 as GST fusion proteins and tested their ability to interact with *in vitro* translated full-length MED1 and RPB1. Interestingly, MED31 showed a strong interaction not only with RPB1, but also with MED1 (Figure 5B), suggesting that MED31 functions as a molecular bridge for MED1–Pol II interaction.

To study the direct role of MED1 phosphorylation in Pol II recycling, we established 293 cell lines stably expressing a single-copy FLAG-tagged WT MED1, a single-copy nonphosphorylatable MED1 mutant (T1032A) or a single-copy phosphomimetic MED1 mutant (T1032D) (Figure 5C). An *in vitro* transcription recycling assay was performed using nuclear extracts from WT or mutant MED1-expressing cell lines. RT-PCR analysis showed that RNA products of the second template recycling were significantly higher in T1032D MED1-expressing cells and lower in T1032A MED1-expressing cells compared to those in WT MED1-expressing cells (Figure 5D), demonstrating that MED1 phosphorylation at T1032 enhances mRNA output during the Pol II recycling process. Western blot analysis further showed that template-bound FLAG-tagged MED1, Pol II (Ser2), total Pol II and MED31 markedly increased in T1032D MED1- and decreased in T1032A MED1-based recycling compared with WT MED1-based recycling (Figure 5E). This indicated that MED1 phosphorylation augments transcription recycling by enhancing recruitment of Pol II (Ser2) and MED31 to the recycling templates. To investigate whether MED31 plays a causal role in transcription recycling, we performed an *in vitro* transcription recycling assay using nuclear extracts from partially MED31-silenced LNCaP-abl cells. MED31 silencing markedly decreased RNA output from transcription recycling as well as MED1 and Pol II levels on the recycling template without affecting MED1 or Pol II protein expression (Figure 5F and G, and Supplementary Figure S3A). Furthermore, MED31 ChIP-seq analysis in LNCaP-abl cells confirmed the genomic distribution of MED31 over class III genes, suggesting that MED31 participates in transcription recycling (Figure 5H and Supplementary Figure S3B). Together, these findings suggest that MED1 phosphorylation promotes an increase in recycled Pol II via MED31, enhancing mRNA output during the Pol II recycling process.

### Pharmacological inhibition of MED1 phosphorylation impairs active transcription, transcription recycling and lethal prostate cancer growth

**Figure 6.**
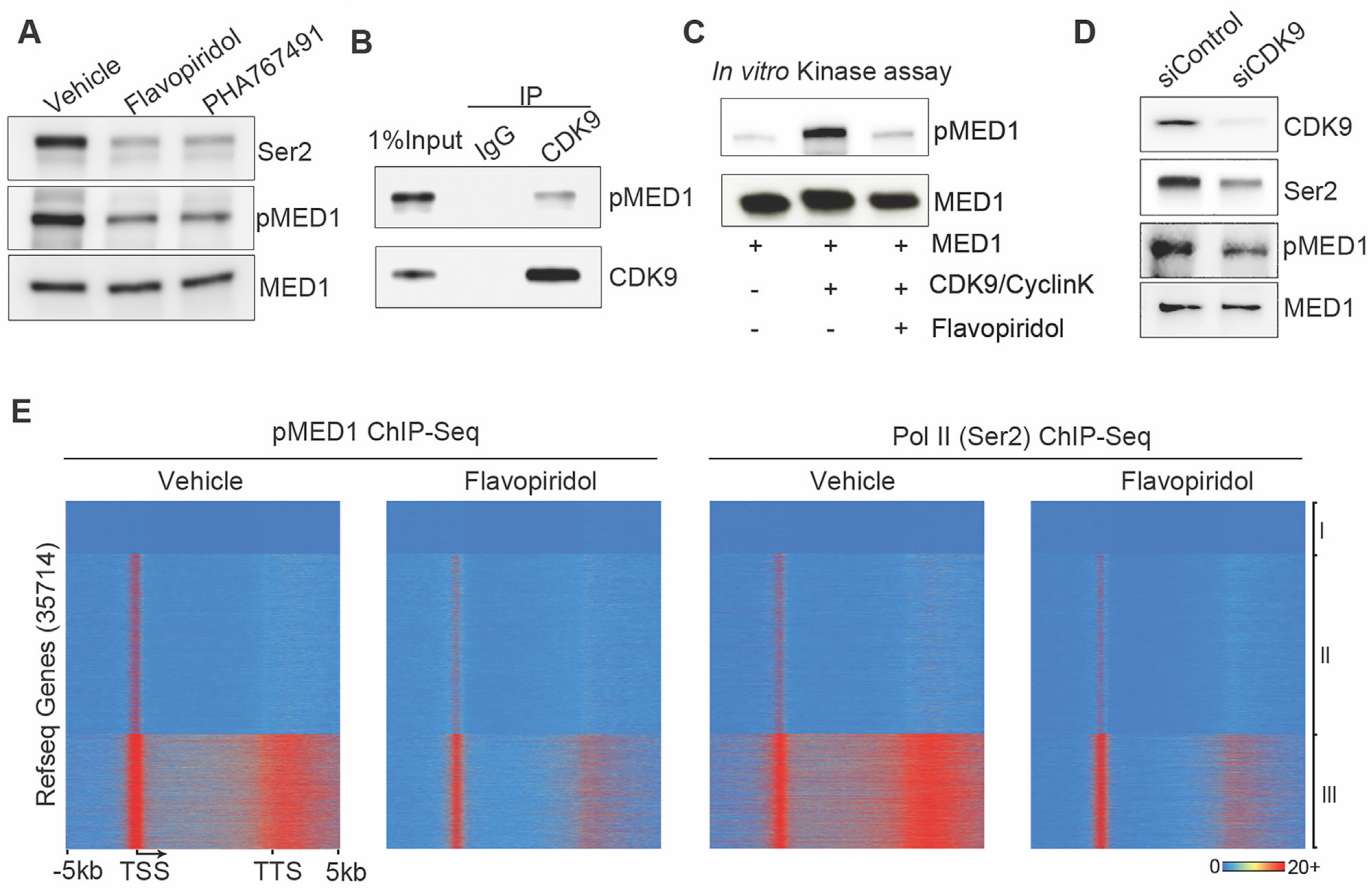
CDK9 phosphorylates MED1 during transcription. **(A)** LNCaP-abl cells were treated with vehicle, FP (1 μM) or PHA767491 (5 μM) for 1 h, and western blot analyses were performed using nuclear extracts with antibodies against Pol II (Ser2), pMED1 or MED1. **(B)** Nuclear extracts were immunoprecipitated with a CDK9 antibody or IgG, and western blot analyses were performed with antibodies against pMED1 and CDK9. **(C)***In vitro* CDK9 kinase assay. Baculovirus recombinant full-length MED1 was incubated with recombinant full-length CDK9 and CyclinK, and western blot analyses were performed. **(D)** LNCaP-abl cells were transfected with siRNA targeting CDK9 or control siRNA for 48 h, and western blot analyses were performed using nuclear extracts with antibodies against CDK9, Ser2 Pol II, pMED1 and MED1. **(E)** Heatmaps show the distribution of ChIP-seq signals of pMED1 and Pol II (Ser2) in LNCaP-abl cells treated for 1 h with vehicle or 1 μM FP.

Having established the causal role of MED1 phosphorylation in driving transcription recycling, we next tested whether pharmacological inhibition of MED1 phosphorylation impairs transcription of actively transcribed class III genes with high levels of Pol II (Ser2) and pMED1 in the gene bodies (Figure 2 and Supplementary Figure S2). Given that FP inhibits productive elongation (38,39) and the serine/threonine kinase CDK9 (38,39,42), and that phosphorylation sites for CDK9 (43) are contained within the T1032 region of MED1, we first examined whether FP treatment inhibits MED1 phosphorylation through inhibition of CDK9. Exposure of LNCaP-abl cells to FP decreased MED1 phosphorylation at T1032 as well as Pol II phosphorylation at Ser2 *in vivo* (Figure 6A). This finding was confirmed by another CDK9 kinase inhibitor, PHA767491 (44) (Figure 6A). As FP also inhibits several other CDKs in addition to CDK9 (45,46), we next asked whether FP inhibition of MED1 phosphorylation is mediated through CDK9. Co-immunoprecipitation experiments showed that CDK9 interacted with pMED1 *in vivo* (Figure 6B). More importantly, *in vitro* kinase assays demonstrated that CDK9 directly phosphorylated a baculovirally expressed full-length MED1 at T1032 and that FP treatment markedly inhibited CDK9-induced phosphorylation (Figure 6C). Moreover, genetic depletion of CDK9 markedly decreased the MED1 phosphorylation in LNCaP-abl (Figure 6D), 22Rv1 and C4-2 CRPC cell models (Supplementary Figure S4A and B) without affecting total MED1 levels. These data indicate that FP can inhibit MED1 phosphorylation at T1032 through inhibition of CDK9. Of note, blocking Pol II elongation by α-amanitin had no effect on MED1 phosphorylation (Supplementary Figure S5A and B), indicating that MED1 phosphorylation by CDK9 occurs upstream of Pol II transcription.

We next examined whether FP treatment inhibits transcription of class III genes. LNCaP-abl cells were treated with FP and ChIP-seq analyses of pMED1 and Pol II (Ser2) were performed in the same population of cells. As expected, treatment of cells with FP for 1 h (38,39) caused a significant decrease in Pol II (Ser2) signals on gene bodies and 3′-end regions of actively transcribed class III genes (Figure 6E), indicating that active transcription was inhibited. Strikingly, FP treatment also caused a significant reduction of pMED1 signals over the same genes in class III (Figure 6E). These results suggest that FP treatment, at least partially through inhibition of CDK9 phosphorylation of MED1, impairs pMED1 binding throughout genes and decreases active transcription.

**Figure 7.**
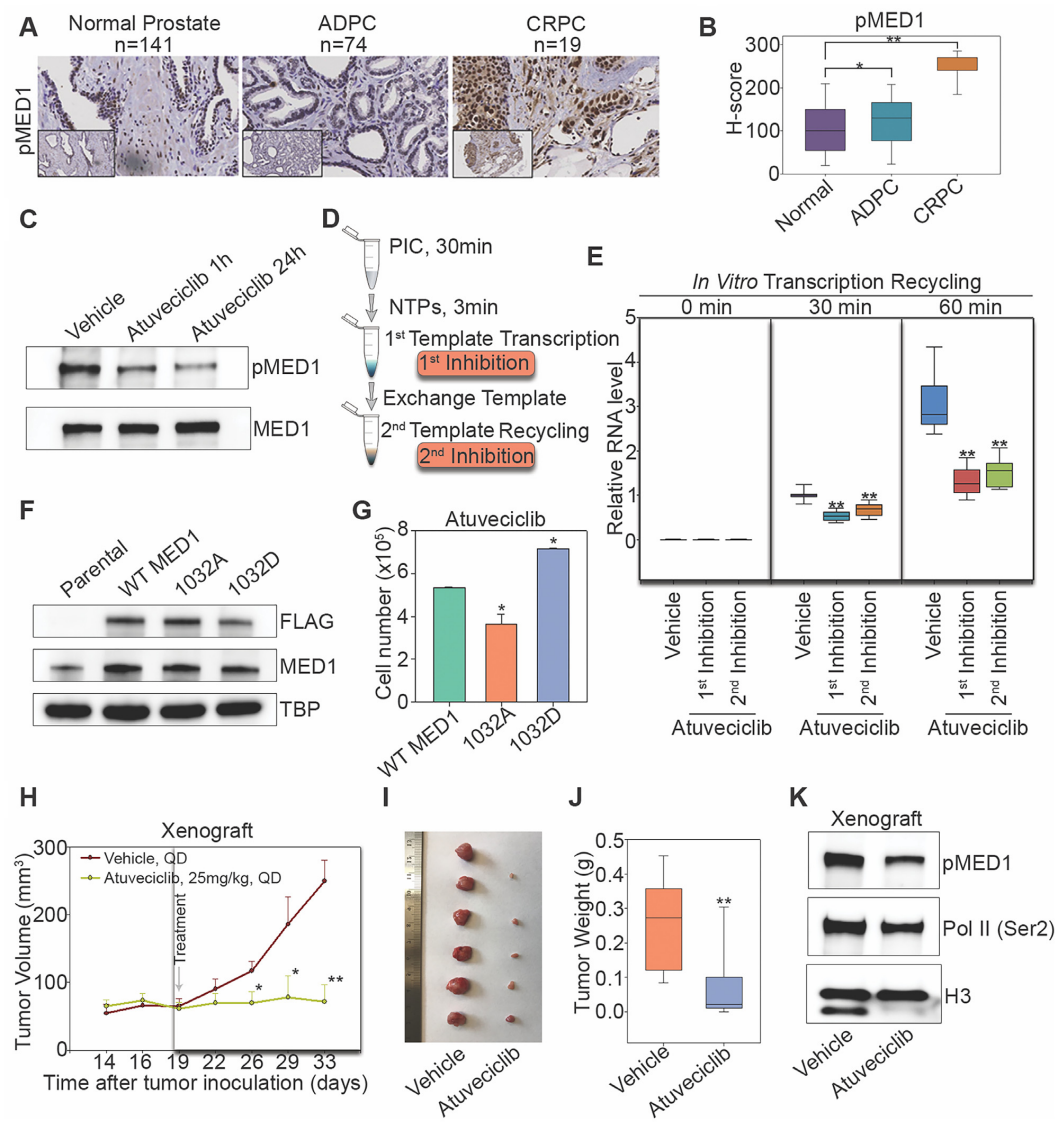
Pharmacological inhibition of pMED1 suppresses transcription recycling and lethal prostate cancer growth. **(A)** Representative pMED1 immunoreactivity in normal prostate, ADPC and CRPC tissues. **(B)** Slides were scanned using an Aperio Digital Pathology Slide Scanner (Leica Biosystems) at 40× magnification and staining was quantified using the Aperio Image Scope (v11). A box plot compares *H*-scores of pMED1 nuclear staining in 234 tissues. The significance was determined by one-way ANOVA, *P < 0.01, **P < 0.001. **(C)** LNCaP-abl cells were treated with vehicle or with atuveciclib (1 μM) for 1 or 24 h. Western blot analyses were performed using nuclear extracts with antibodies against pMED1 and MED1. **(D)** Schematic of the drug-inhibited transcription recycling assay. Atuveciclib (1 μM) was added either during first template transcription after 3 min of NTP addition or during second template recycling after exchange of template. **(E)** RNA products from the second template reaction were analyzed by reverse transcription followed by qPCR quantification. Regions = 7 (*n* = 2), *P*-values were calculated using two-tailed Student’s *t*-test, *P < 0.01, **P < 0.001. **(F)** Western blots of lysates from LNCaP-abl cells transiently expressing WT or mutated FLAG-MED1 proteins. **(G)** Cell proliferation with atuveciclib treatment was measured by direct cell counting assays. Results are mean ± SD of duplicate experiments. The significance was determined by one-way ANOVA, *P < 0.05. **(H)** Tumor growth in mice treated with atuveciclib (*n* = 11) or vehicle (*n* = 11) once daily. Treatments were started 19 days after inoculation and continued for 14 days. Data are represented as mean ± SEM. Significance was calculated using two-tailed Student’s *t*-test, *P < 0.05, **P < 0.001. **(I)** Representative picture of vehicle- and atuveciclib-treated LNCaP-abl tumors at time of collection. **(J)** Tumor weight for vehicle (*n* = 11) and atuveciclib (*n* = 11) groups. The significance was determined by two-tailed Student’s *t*-test, **P < 0.001. **(K)** Western blots were performed using nuclear extracts from pooled xenograft tissues from vehicle- (*n* = 2) or atuveciclib-treated mice (*n* = 6) with the indicated antibodies.

Finally, we asked whether pharmacological inhibition of MED1 phosphorylation can be used to treat pMED1-relevant diseases. As our previous studies found that MED1 phosphorylation is markedly higher in LNCaP-abl CRPC cell model than in LNCaP (a cell model for earlier phase ADPC) (23), we first performed immunocytochemical analysis of pMED1 in normal prostate, ADPC and CRPC patient tissues. We found that the CRPC samples had significantly higher staining of pMED1 than the ADPC and normal prostate samples (Figure 7A and B), suggesting that MED1 phosphorylation increases during prostate cancer progression to the lethal phase. Next, we analyzed TCGA prostate cancer data (47) and found that the expression of *CDK9* and *MED1* was positively correlated in prostate cancer (Supplementary Figure S6A). Moreover, high *CDK9* expression was associated with poor prognosis of prostate cancer patients (Supplementary Figure S6B). These results support a potential involvement of CDK9 in modifying MED1 in prostate cancer. Although the existing small molecule FP is effective in inhibition of CDK9, it is highly toxic and leads to severe side effects in patients (46). In addition, FP is not a CDK9-specific inhibitor (46). We thus examined the effect of atuveciclib (BAY 1143572), the first highly selective CDK9 inhibitor that has entered clinical trials but has not yet been used to treat CRPC patients (25,48), on MED1 phosphorylation, transcription recycling and CRPC cell growth. Atuveciclib treatment markedly decreased MED1 phosphorylation in LNCaP-abl and 22Rv1 CRPC cells (Figure 7C and Supplementary Figure S6E), and the IC_50_ value of atuveciclib for inhibition of LNCaP-abl and 22Rv1 cell growth was ∼0.5 and 2.1 μM, respectively (Supplementary Figure S6C and D). Further RNA-seq analysis revealed that atuveciclib preferentially inhibited the expression of class III genes (Supplementary Figure S6F). We then investigated whether atuveciclib could inhibit transcription recycling using a modified drug-inhibited transcription recycling assay (Figure 7D). In this assay, atuveciclib was introduced in two different ways. In the first experiment, atuveciclib was added after transcription started on the first template and maintained for the rest of recycling assay. In the second experiment, atuveciclib was added only during the second template recycling. Both types of inhibition significantly decreased RNA products from the second template recycling (Figure 7E), demonstrating that CDK9 inhibition impairs transcription recycling. To examine whether atuveciclib inhibits cell growth in a pMED1-specific manner, we measured cell growth in WT MED1-, MED1 T1032D- or MED1 T1032A-overexpressing LNCaP-abl and 22Rv1 cells treated with or without atuveciclib. Compared to T1032A, T1032D showed significant resistance to atuveciclib-induced growth inhibition (Figure 7F and G, and Supplementary Figure S6G and H), suggesting that atuveciclib decreases CRPC cell growth at least partially through downregulation of MED1 phosphorylation. We finally examined the effect of atuveciclib on CRPC tumor growth in mice. Daily oral administration of atuveciclib resulted in a significant decrease in tumor volumes and tumor weights compared to vehicle groups (Figure 7H–J and Supplementary Figure S6I), whereas <10% of body weight change was observed (Supplementary Figure S6J). Notably, western blot analysis of engrafted tumor tissues showed that atuveciclib markedly decreased MED1 phosphorylation (Figure 7K). These data suggest that inhibition of MED1 phosphorylation by CDK9 inhibitor can reduce transcription recycling and decrease lethal prostate cancer growth.

## DISCUSSION

While transcription processes such as initiation and elongation have been well studied, transcription recycling is an overlooked but essential transcription process that contributes to overall transcriptional output. It is poorly understood how transcription recycling is regulated. Using an integration of *in vitro* transcription recycling analysis with static/dynamic ChIP-seq analysis, we found that pMED1 dynamically travels with Pol II (Ser2) during transcription recycling (Figures 1–4). Further functional studies on WT MED1 and MED1 phospho-mutants demonstrate that phosphorylation of MED1 drives transcription recycling and enhances mRNA output (Figure 5).

It remains an open question whether mammalian Mediator is able to enter gene bodies to regulate Pol II transcription. Previous studies have found that MED26 and CDK8 do not globally travel with Pol II (8). Our observation that MED16 and MED17 exhibit no decrease on the first template from the PIC state to the multi-round transcription state (Figure 1E) similarly suggests that these two subunits might not travel along the transcription templates. In contrast, our *in vitro* and *in vivo* analyses have shown that MED1, when phosphorylated at T1032 by CDK9 (Figure 6A–D), dynamically travels with Pol II and drives Pol II transcription recycling (Figures 2–5 and Supplementary Figure S7). Interestingly, mechanistic studies have found that MED31 functions as a molecular bridge for MED1–Pol II interaction, and is causally linked to transcription recycling (Figure 5). Previous structural studies on *S. pombe* and *S. cerevisiae* have revealed close contacts between Med31 and Pol II CTD (40,41). Future structural analysis will help to further elucidate the interaction and crosstalk among Pol II, MED1 and MED31, as well as other Mediator subunits.

Although previous studies have found that PI3K/AKT phosphorylates MED1 in prostate cancer cells (23,49), AKT1 and AKT2 are mainly localized outside of the nucleus (50). These data imply that MED1 may be further or *de novo* phosphorylated by other kinases in the nucleus and/or on chromatin. Interestingly, we have found that CDK9 interacts with and phosphorylates MED1 in the nucleus (Figure 6). Similar to the case of c-Myc (13), CDK9-phosphorylated MED1 binds to almost all actively transcribed genes (Figures 2B and 6E, and Supplementary Figure S2). Furthermore, CDK9 inhibition-induced MED1 dephosphorylation contributes to FP-induced global inhibition of active transcription (Figure 6). Since MED1 phosphorylation is significantly increased during prostate cancer progression (Figure 7A), we proposed that pharmacological inhibition of CDK9 can block MED1 phosphorylation, transcription recycling and CRPC growth. Indeed, CDK9 inhibition by atuveciclib (BAY 1143572), a highly selective CDK9 inhibitor (25,48), significantly decreases CRPC cell growth in culture and in mice through inhibition of MED1 phosphorylation and transcription recycling. Previous studies have found that targeting CDK9 in cancers can inhibit oncogenes (e.g. *MYC*) (45) and reactivate tumor suppressor genes (e.g. *CHD5*) (51). Our findings that targeting CDK9 inhibits pMED1-driven transcription recycling and CRPC growth provide novel mechanistic insight into the known anticancer activities of CDK9 inhibitors. Since clinical studies of CDK9 inhibitors have been somewhat disappointing partially, due in part to a lack of biomarkers of pharmacological efficacy (9), future studies should investigate whether MED1 phosphorylation can serve as a biomarker for predicting CDK9 inhibitor therapeutic response in cancers.

## DATA AVAILABILITY

High-throughput sequencing data have been deposited in the Gene Expression Omnibus database under accession number GSE127894. Proteomics data have been deposited in the PRIDE database under accession number PXD017881.

## Supplementary Material

gkac246_Supplemental_Files
