# Supplementary material for: Phosphorylated MED1 links transcription recycling and cancer growth
Source: Nucleic Acids Res. 2022 Apr 8;50(8):4450–63. doi: 10.1093/nar/gkac246 (PMC9071494; doi:10.1093/nar/gkac246)
Supplement: gkac246_Supplemental_Files [file gkac246_supplemental_files.zip › Final 030922_pMED1 manuscript Supplementary Materials.pdf]

Phosphorylated MED1 links transcription recycling and cancer growth

Zhong Chen[1,7,*], Zhenqing Ye[2,7], Raymond E. Soccio[3], Tomoyoshi Nakadai[4], William Hankey[1], Yue Zhao[1], Furong Huang[1], Fuwen Yuan[1], Hongyan Wang[1], Zhifen Cui[1], Benjamin Sunkel[5], Dayong Wu[5], Richard K. Dzeng[3], Jennifer M. Thomas-Ahner[6], Tim H.M. Huang[2], Steven K. Clinton[6], Jiaoti Huang[1], Mitchell A. Lazar[3], Victor X. Jin[2,*], Robert G. Roeder[4], and Qianben Wang[1,*]

Supplementary Figures S1-S7

Supplementary Materials and Methods

Figure S1

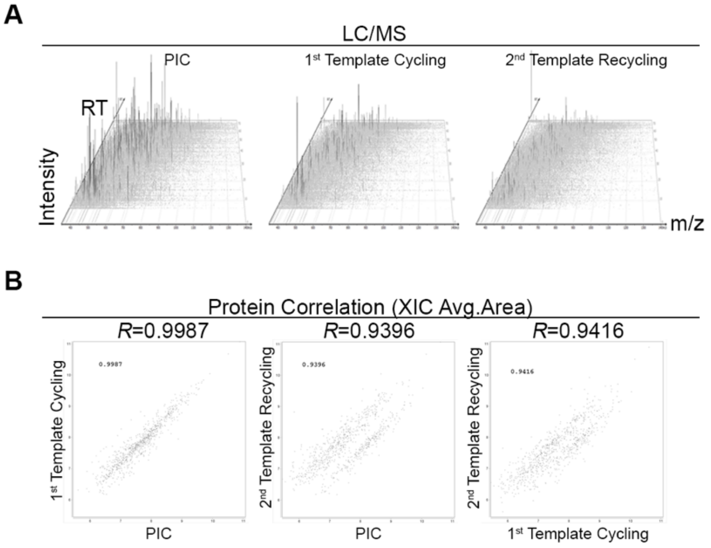

C

Mediator Subunits in Multi-round Transcription

Protein	-10lgP	#Peptides	#Unique
MED1	233.2	55	55
MED12	221.55	60	56
MED14	208.06	46	46
MED24	203.27	36	35
MED23	202.98	41	41
MED13	176.44	26	26
MED17	170.53	25	25
MED16	170.37	20	20
MED27	155.45	14	14
MED15	137.01	13	13
MED4	136.52	12	12
MED8	135.89	10	10
CDK8	122.62	12	6
MED29	118.89	7	7
MED25	116.5	9	9
MED20	108.35	7	7
MED9	108	6	6
MED6	106.33	7	7
MED10	106.28	5	5
MED19	105.09	5	5
MED7	103.92	5	5
MED31	100.89	6	6
MED22	98.12	7	7
MED30	85.81	6	6
MED11	84.91	3	3
MED26	71.98	6	6
MED28	62.22	3	3

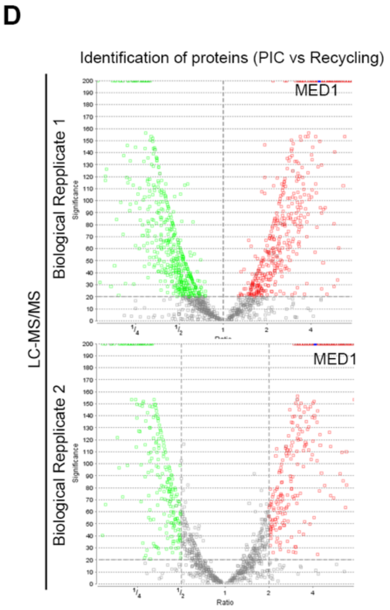

Supplementary Figure S1. LC-MS/MS analysis of template-bound proteins. **(A)** Intensity view of LC-MS/MS. **(B)** Correlation of proteins identified in PIC and multi-round transcription phases as well as recycling phase on the 2nd template. **(C)** Template-bound proteins identified by LC-MS/MS in multi-round transcription on 1st template. **(D)** Volcano Plot displaying differential proteins enriched in recycling relative to PIC in two independent biological replicates.

Figure S2

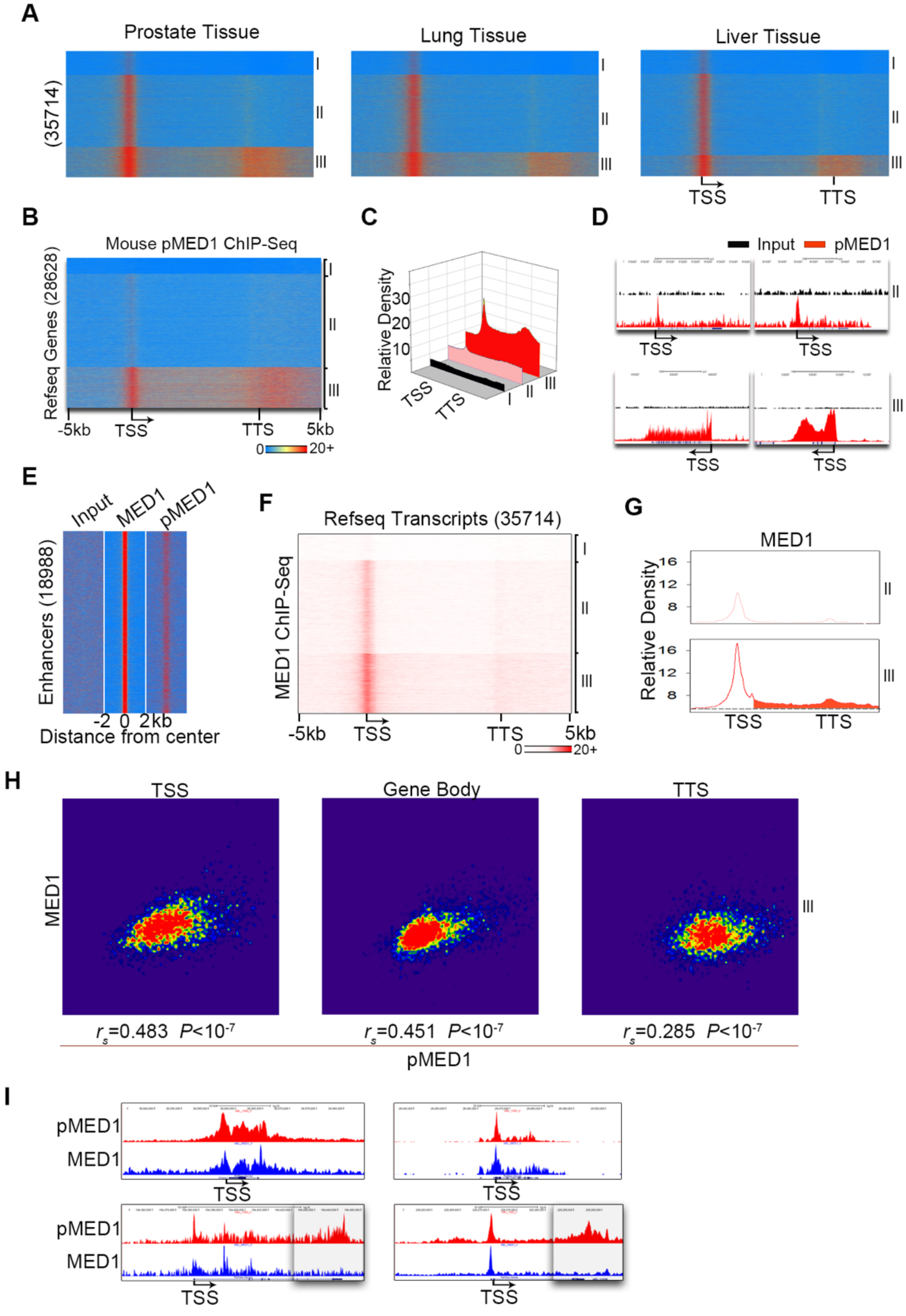
A
Prostate Tissue
Lung Tissue
Liver Tissue
(35714)
I
II
III
TSS
TTS
B
Mouse pMED1 ChIP-Seq
Refseq Genes (28628)
-5kb
TSS
TTS
5kb
0
20+
C
Relative Density
30
20
10
TSS
TTS
I
II
III
D
Input
pMED1
E
Input
MED1
pMED1
Enhancers (18988)
-2
0
2kb
Distance from center
F
Refseq Transcripts (35714)
MED1 ChIP-Seq
G
MED1
Relative Density
16
12
8
H
TSS
Gene Body
MED1
r_s=0.483 P<10^{-7}
r_s=0.451 P<10^{-7}
r_s=0.285 P<10^{-7}
pMED1
I
pMED1
MED1

Figure S2

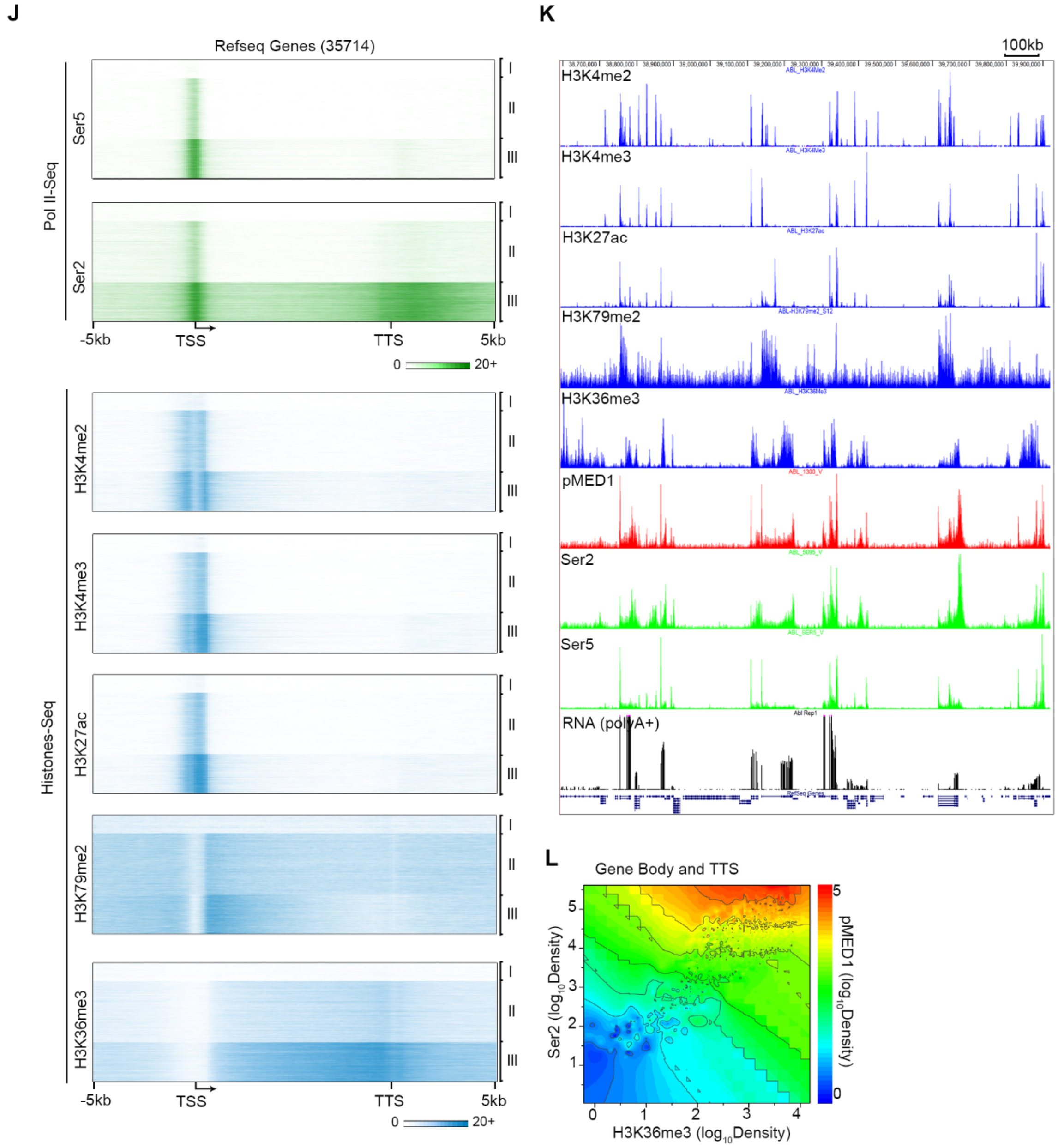

J
Refseq Genes (35714)
Pol II-Seq
Ser5
Ser2
I
II
III
-5kb
TSS
TTS
5kb
0
20+
Histones-Seq
H3K4me2
H3K4me3
H3K27ac
H3K79me2
H3K36me3
K
100kb
H3K4me2
H3K4me3
H3K27ac
H3K79me2
H3K36me3
pMED1
Ser2
Ser5
RNA (polyA+)
L
Gene Body and TTS
Ser2 (log10Density)
H3K36me3 (log10Density)
pMED1 (log10Density)

Figure S2

M

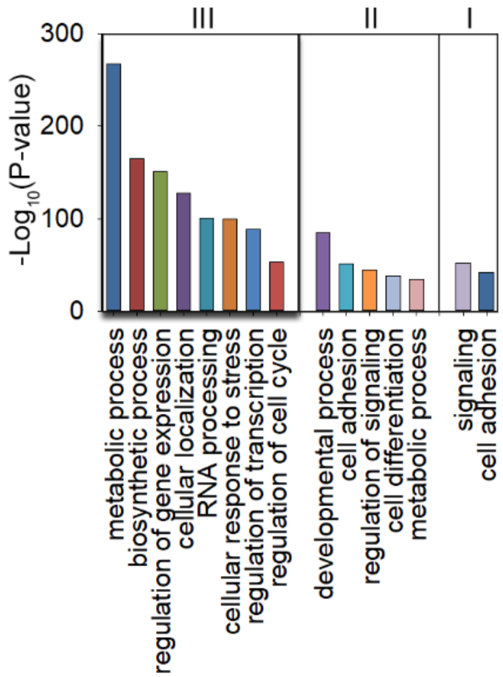

Supplementary Figure S2. Genomic distribution and function of pMED1 in mouse and human genomes. **(A)** Heatmaps illustrate pMED1 tissue ChIP-seq signals over RefSeq genes in human prostate, liver, and lung tumors, respectively. **(B)** A heatmap shows the distribution of pMED1 ChIP-seq signals on scaled RefSeq genes in mouse 3T3-L1 adipocytes. **(C)** Average pMED1 signal densities along the three classes of scaled mouse RefSeq genes. **(D)** UCSC Genome Browser views of pMED1 binding to representative class II/III genes in mouse 3T3-L1 adipocytes. **(E)** Heatmaps show input, MED1 and pMED1 ChIP-seq (column) signal densities on 18,988 enhancer regions which were defined by MACS2 using MED1 ChIP-seq data (rows). **(F)** A heatmap shows the distribution of MED1 ChIP-seq signals on scaled Refseq genes (rows). **(G)** Average MED1 ChIP-seq signal densities on scaled class II and III genes. **(H)** Correlation of MED1 and pMED1 ChIP-seq signal densities on transcription start site (TSS) regions, gene bodies, and 3'-end peak regions (TTS), respectively. **(I)** UCSC Genome Browser views showing binding similarities and differences between pMED1 and MED1 across the gene bodies and 3'-end regions of 4 representative genes. The difference between 3'-end signals of pMED1 and MED1 is highlighted. **(J)** Heatmaps show the distribution of ChIP-seq signals of Pol II (Ser5), Pol II (Ser2), H3K4me2, H3K4me3, H3K27Ac, H3K79me2 and H3K36me3 over the three classes of genes in human LNCaP-abl cells. **(K)** An example of genomic distributions of 5 histone marks as well as pMED1, Pol II (Ser2), Pol II (Ser5), and the mRNA expression over a 1.5 Mb region containing multiple class III genes. **(L)** Correlation of ChIP-seq signal density within gene body and 3'-end regions based on H3K36me3, Pol II (Ser2) and pMED1 ChIP-seq data. 3D matrix was generated from kriging correlation. **(M)** Gene Ontology (GO) analysis of three classes of genes.

Figure S3

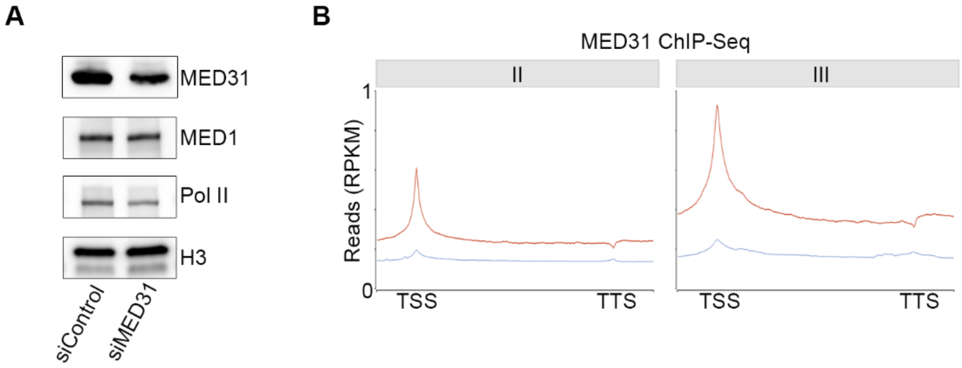

Supplementary Figure S3. (**A**) LNCaP-abl cells were transfected with siControl or siMED31. Seventy-two h after siRNA transfection, western blot analyses were performed using indicated antibodies. (**B**) Average MED31 ChIP-seq signal densities over the scaled human RefSeq genes of class II and III in LNCaP-abl cells.

Figure S4

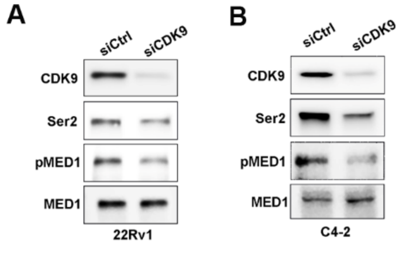

Supplementary Figure S4. Genetic inhibition of CDK9 decreases MED1 phosphorylation in CRPC cells. 22Rv1 (**A**) and C4-2 (**B**) cells were transfected with siRNA targeting CDK9 or control siRNA for 48 h, and western blot analyses were performed using nuclear extracts with antibodies against CDK9, Ser2 Pol II, pMED1 and MED1.

Figure S5

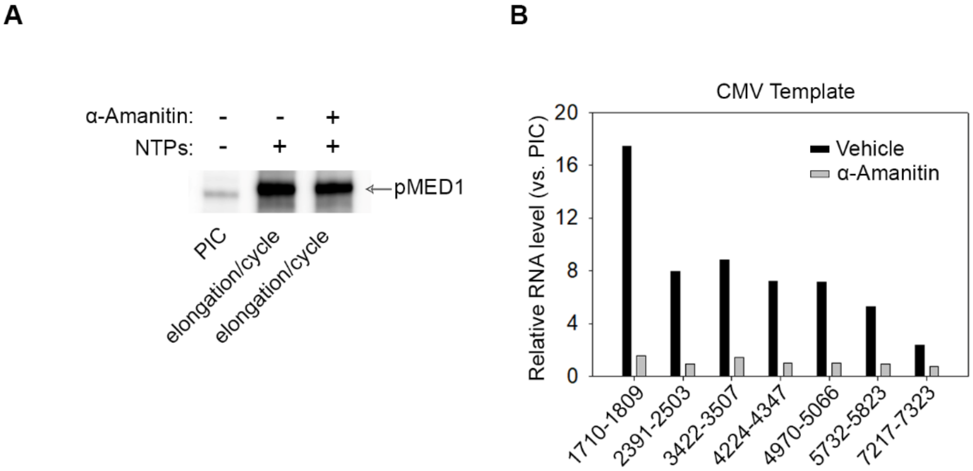

Supplementary Figure S5. MED1 phosphorylation is not dependent on Pol II transcription. 100 ng of CMV template was incubated with 400 μg of HeLa nuclear extract for 30 min to form PIC. NTPs were then added and incubated for 3 min to allow activation of some factors (e.g. phosphorylation of MED1 by CDK9) and start of elongation. Next, 10 μM of α-amanitin was added in the reaction to block Pol II elongation. After 30 min, all reactions were stopped by addition of 25 mM EDTA. **(A)** pMED1 western blot analysis of samples treated with or without α-amanitin in PIC and elongation/cycle steps. **(B)** RT-qPCR analysis of RNA generated from the CMV template in the presence or absence of α-amanitin.

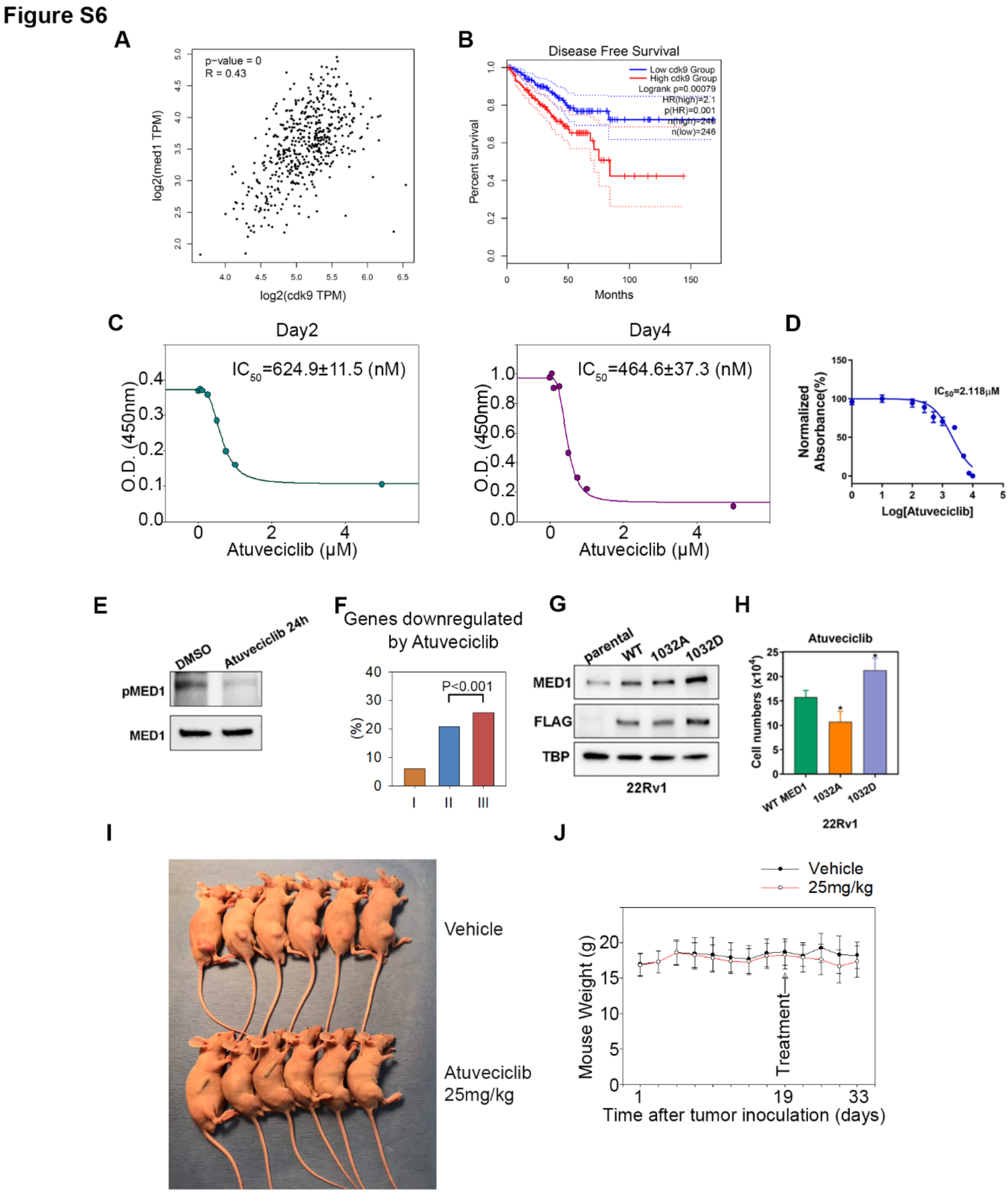

Supplementary Figure S6. Atuveciclib inhibits lethal prostate cancer growth *in vitro* and *in vivo*. **(A)** Corelation of *CDK9* and *MED1* expression in TCGA prostate cancer dataset. **(B)** Survival analysis of *CDK9* expression in TCGA prostate cancer dataset (dbGaP Study Accession: phs000178.v11.p8) (1). **(C)** IC_{50} was determined in LNCaP-abl cells using WST-1 assay. **(D)** IC50 was determined in 22Rv1 cells using the WST-1 assay. **(E)** 22Rv1 cells were treated with vehicle or with Atuveciclib (3 uM) for 24 h. Western blot analyses were performed using nuclear extracts with antibodies against pMED1 and MED1. (**F**) Data represents the ratio of all three class genes

downregulated by Atuveciclib for 24 h by RNA-seq. **(G)** Western blots of lysates from 22Rv1 cells transfected with a WT or a mutated FLAG-MED1. **(H)** 22Rv1 cell proliferation with Atuveciclib treatment was measured by direct cell count assays. Results are mean ± SD of duplicate experiments. The significance was determined by one-way ANOVA, $^*p < 0.05$. **(I)** Images of the dorsal flank containing subcutaneous xenografts from six representative animals per group. **(J)** Average body weights for vehicle (n=11) and Atuveciclib treatment groups (n=11) of mice. The results are shown as mean ± SD.

Figure S7

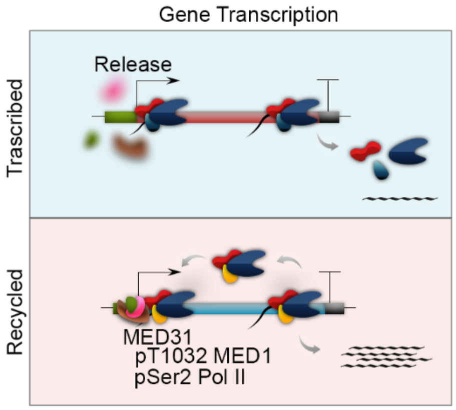

Supplementary Figure S7. CDK9-phosphorylated MED1 (pT1032 MED1) dynamically travels with Ser2 phosphorylated Pol II throughout the transcribed genes to drive Pol II recycling. MED31 facilitates pMED1 and Pol II recycling as a molecular bridge.

Supplementary Materials and Methods

In vitro transcription recycling assay

Nuclear protein extraction. Nuclear extract used in this system was prepared as described with modifications (2). Briefly, 293 or LNCaP-abl cells were washed, trypsinized and resuspended in cold PBS. After centrifugation, cell pellets were suspended in ten volumes of Buffer A (10 mM HEPES pH 7.9, 3 mM MgCl2, 10 mM KC1 and 0.5 mM DTT) and allowed to stand for 10 min. All cells were lysed by 10 strokes of Dounce homogenizer (B type pestle) and checked by Trypan blue staining. Nuclei were collected after centrifugation for 10 min at 4,000 rpm at 4ºC and resuspended in two volumes of Buffer C (20 mM HEPES pH 7.9, 25% (v/v) glycerol, 0.42 M NaCl, 1.5 mM MgCl2, 0.2 mM EDTA, 0.5 mM DTT and 1x Protease Inhibitor Cocktail) for 40 min at 4ºC. The suspension was centrifuged for 20 min at 25,000 x g and dialyzed against 100 volumes of Buffer D (20 mM HEPES pH 7.9, 20% (v/v) glycerol, 0.1 M KCl, 0.2 mM EDTA, 0.5 mM DTT, 1x Protease Inhibitor Cocktail) for 6-12 h. The supernatant was frozen in aliquots on dry ice and stored at -80 ºC. The protein concentration was usually 2 to 5 mg per ml.

Linear DNA template amplification and immobilization. Linearized DNA templates were prepared by the amplification of p300 and pEF1 plasmids using 5'-biotinylated forward primers with Q5 High-Fidelity 2X Master Mix following manufacturer's instructions. The CMV template (from p300) includes the CMV enhancer (235-614), the CMV promoter (615-818), and the ORF (1020-8279). The EF1α Template (from pEF1) consists of the EF-1α promoter (2013-3191) and the ORF (3207-5615). Initial denaturation was performed at 98°C for 30 seconds, followed by 30 cycles of 98°C for 10 seconds, 56°C for 30 seconds, and 72°C for 10 min, with final extension performed at 72°C for 10 minutes. Amplified templates were purified using AMPure XP beads

and quantified with a DeNovix DS-11 FX+ spectrophotometer (Wilmington, DE). The Dynabeads kilobaseBINDERTM Kit was used for immobilizing long double-stranded DNA templates. 60 pmoles of biotinylated DNA fragments were immobilized onto 1 mg Dynabeads M-280 Streptavidin or Pierce™ NeutrAvidin™ Agarose (Thermo) for 3 h at room temperature. After washing in 10 mM Tris-HCl pH 7.5, 1 mM EDTA, and 2.0 M NaCl twice and Tris-HCl pH 8.0 three times, immobilized templates were resuspended in distilled RNase-free water.

***In vitro* transcription and recycling.** For transcription on the 1st template, PIC formation was set up by incubating 3-5 mg of nuclear proteins with 100 pmoles of immobilized template in a final volume of 1.2 ml (10 mM HEPES pH 7.9, 10% (v/v) glycerol, 0.25 mM DTT, 0.1 mM EDTA, 50 mM KCI, 3 mM $MgCl_2$) for 30 min at 30°C at 1000 rpm. The transcription was started by addition of NTP mix to a final concentration of 600 μM each, and allowed to proceed for 1 h. The reaction was then stopped by placing the tube on a magnet for 30 seconds followed by discarding the supernatant. Templates were washed quickly twice for 2 min in ice-cold washing buffer (10 mM HEPES pH 7.9, 50 mM KCl, 0.1 mM EDTA, 0.25 mM DTT 10% glycerol, 0.05% NP-40) and once without NP-40. Transcription on the 1st template was allowed to restart by incubating templates with new transcription buffer (10 mM HEPES pH 7.9, 10% (v/v) glycerol, 0.25 mM DTT, 0.1 mM EDTA, 50 mM KCI, 3 mM $MgCl_2$, 600 μM NTP mix). After 30 min, the 1st round of solutions from the 1st template were collected by separating the 1st template-coated beads with a magnet for 30 seconds. New transcription buffer was added back to the 1st template-coated beads and transcription was allowed to proceed for another 30 min before the 2nd round of transcription solutions from the 1st template were collected. The 1st or 2nd round of solutions from the 1st template were incubated immediately with the 2nd template to allow transcription recycling start. After incubating for 30 min and 1 h at 30°C, solutions containing the 2nd template-derived RNA product

were collected for RNA quantification. Proteins bound to the 2nd template were eluted in 1x SDS loading buffer and analyzed by gel electrophoresis. Of note, "1st template" and "2nd template" are defined differently in the gene expression analysis as opposed to the proteomics analysis. For analysis of RNA transcripts from recycling, we used the CMV template as "1st template" and the EF1α Template as "2nd template", as the RNA products from the 2nd template (i.e. RNA from recycling) must be distinguishable from those from the 1st template. For proteomic analysis of proteins associated with the templates, we used the same CMV template as "1st template" in PIC and multi-round transcription steps and "2nd template" in the recycling step. Using the sample template (with the same promoter) helps avoid promoter bias when we compare associated proteins from different transcription steps.

RNA quantification by qPCR. RNA quantification of in vitro transcription was performed as described with some modifications (3). RNA products in transcription recycling were purified using the RNeasy mini kit with On-Column DNase digestion (Qiagen), and further concentrated using the RNeasy MinElute Cleanup kit. Next, reverse transcription was performed with SuperScript IV VILO Master Mix with additional ezDNase enzyme treatment (Thermo). The DNA digestion reaction was incubated at 37°C for 5 min followed by annealing at 25°C for 10 min, reverse transcription of RNA at 50°C for 10 min and inactivation of enzyme at 85°C for 5 min. The diluted cDNA was then used for qPCR quantification (Thermo). Primer pairs (4) were designed for qPCR detection to cover the transcribed region of the pEF1 template. Standard curves were generated for each primer pair and used to calculate RNA copy number. By normalizing to the copy number of one reaction (vehicle or siControl or wild type) on the 2nd template at 30 min, relative RNA level was finally determined for each experiment.

Two-dimensional difference gel electrophoresis (2D-DIGE)

2-D DIGE was performed by Applied Biomics, Inc (Hayward, CA). Briefly, proteins bound to DNA template were eluted in 2D lysis buffer (7 M urea, 2 M thiourea, 4% CHAPS, 30 mM Tris-HCl, pH 8.8), and protein concentration was measured by the Bio-Rad protein assay method. For protein labeling, 30 μg of each protein sample was labeled with 0.7ml of Cy2, Cy3, or Cy5 at 4°C for 30 min. Labeling was then stopped by the addition of 0.7ml of 10 mM L-Lysine and incubation for 15 min at 4°C. The labeled samples were mixed together and diluted with an equal volume of 2x 2-D sample buffer (8 M urea, 4% CHAPS, 20 mg/mL DTT, 2% pharmalytes, and trace amount of bromophenol blue), 100 ml destreak solution. After adjusting the final volume with rehydration buffer (7 M urea, 2 M thiourea, 4% CHAPS, 20 mg/mL DTT, 1% pharmalytes, and trace amount of bromophenol blue), samples were incubated at room temperature for 10 min and centrifuged for 10 min at 16,000 x g, and then subjected to isoelectric focusing (pH 3-10) according to the GE Healthcare protocol. The IPG strips were incubated in fresh equilibration buffer #1 (50 mM Tris-HCl, pH 8.8, containing 6 M urea, 30% glycerol, 2% SDS, trace amount of bromophenol blue and 10mg/ml DTT) for 15 min and subsequently rinsed in fresh equilibration buffer #2 (50 mM Tris-HCl, pH 8.8, containing 6 M urea, 30% glycerol, 2% SDS, trace amount of bromophenol blue, and 45 mg/ml lodoacetamide) for 10 min with gentle agitation. IPG strips were rinsed once in the SDS-gel running buffer and transferred to 8% SDS-Gels, and run at 150V at 15°C until the dye front reached the bottom of the gel. After SDS-PAGE, gels were scanned using the Typhoon TRIO (GE Healthcare, Waukesha, WI) following the manufacturer's instructions. The images were analyzed and processed by Image Quant software (version 6.0, GE Healthcare, Waukesha, WI), and quantitation analysis was done with DeCyder software (version 6.5, GE Healthcare, Waukesha, WI).

Nano-liquid chromatography coupled with tandem mass spectrometry (Nano-LC-MS/MS Analysis)

In this experiment, all protein samples were subjected to digestion with trypsin, and were analyzed on a Dionex Ultimate 3000 Nano LC system coupled with an Obitrap Q Exactive HF mass spectrometer (Thermo Fisher Scientific, USA) with an ESI nanospray source by Creative Proteomics (Shirley, NY). Briefly, the samples were reduced by 10 mM DTT at 56°C for 1 h and alkylated by 20 mM IAA at room temperature in the dark for 1 h. Free trypsin was added into the protein solution at a ratio of 1:50, and the solution was incubated at 37°C overnight. Samples were cleaned up with C18 tips, and the extracted peptides were lyophilized to near dryness. Peptides were resuspended in 2-20 µl of 0.1% formic acid before LC-MS/MS analysis. 5 µl of sample was loaded onto a 100 µm×10 cm in-house made column packed with a reversed-phase ReproSil-Pur C18-AQ resin (3 µm, 120 Å, Dr. Maisch GmbH, Germany). Peptides were separated at an analytical flowrate of 600 nl/min with linear gradient (A: 0.1% formic acid in water; B: 0.1% formic acid in acetonitrile): from 6% to 9% B for 8 min, from 9% to 14% B for 16 min, from 14% to 30% B for 36 min, from 30% to 40% B for 15 min and from 40% to 95% B for 3 min, eluting with 95% B for 7 min. Data dependent acquisition was performed using the in positive ion mode. Survey spectra were acquired in the Orbitrap with a resolution of 60,000 and a mass range from 300 to 1800 m/z. Up to 20 of the most intense ions from the preview scan in the Orbitrap were isolated, fragmented and analyzed in the LTQ part of the instrument. MS files were analyzed and searched against a human protein database based on UniProtKB/Swiss-Prot (2018) using Peaks Studio 10.0 (Bioinformatics Solutions, Canada). The parameters were set as follows: the protein modifications were carbamidomethylation (C) (fixed), oxidation (M) (variable), deamidation (NQ) (variable); the enzyme specificity was set to trypsin; the maximum missed cleavages were set to

2; the precursor ion mass tolerance was set to 10 ppm, and MS/MS tolerance was 0.5 Da. Only high confidence identified peptides (FDR 1%) were chosen for downstream protein identification analysis.

Antiboies used

M2-FLAG Sigma-Aldrich Cat#F1804

MED1 Santa Cruz Biotechnology Cat#sc-8998

MED1 Santa Cruz Biotechnology Cat#sc-5334

MED1 Bethyl Cat#A300-793A

pMED1 Yenzym N/A

MED16 Novus Biologicals Cat#NBP2-04141

MED17 ThermoFisher Cat#PA5-40839

MED31 Novus Biologicals Cat# H00051003-M01

MED31 ThermoFisher Cat# PA5-70247

CDK9 Santa Cruz Biotechnology Cat#sc-484

TBP Santa Cruz Biotechnology Cat#sc-204

Pol II (8WG16) Abcam Cat#ab817

Pol II (phospho S5) Abcam Cat#ab5131

Pol II (phospho S2) Abcam Cat#ab5095

H3K4me2 Abcam Cat#ab7766

H3K4me3 Abcam Cat#ab8580

H3K27ac Abcam Cat#ab4729

H3K36me3 Abcam Cat#ab9050

H3K79me2 Abcam Cat#ab3594

H3 Abcam Cat#ab209023

TBP Abcam Cat#ab197874

Goat anti-mouse IgG (H+L) secondary antibody, HRP Azure biosystems Cat#AC2115

Goat anti-rabbit IgG (H+L)

Secondary antibody, HRP Azure biosystems Cat#AC2114

Donkey anti-goat IgG-HRP Santa Cruz Biotechnology Cat# sc-2020

Supplementary References

1. Tang, Z., Li, C., Kang, B., Gao, G., Li, C. and Zhang, Z. (2017) GEPIA: a web server for cancer and normal gene expression profiling and interactive analyses. *Nucleic Acids Res*, **45**, W98-W102.
2. Dignam, J.D., Lebovitz, R.M. and Roeder, R.G. (1983) Accurate transcription initiation by RNA polymerase II in a soluble extract from isolated mammalian nuclei. *Nucleic Acids Res*, **11**, 1475-1489.
3. Voss, C., Schmitt, B., Werner-Simon, S., Lutz, C., Simon, W. and Anderl, J. (2014) A novel, non-radioactive eukaryotic in vitro transcription assay for sensitive quantification of RNA polymerase II activity. *BMC Mol Biol*, **15**, 7.
4. Chen, Z., Hankey, W., Zhao, Y., Groth, J., Huang, F., Wang, H., Campos, A.R., Huang, J., Roeder, R.G. and Wang, Q. (2021) Transcription recycling assays identify PAF1 as a driver for RNA Pol II recycling. *Nat Commun*, **12**, 6318.
