# Supplementary material for: Phosphorylated MED1 links transcription recycling and cancer growth
Source: Nucleic Acids Res. 2022 Apr 8;50(8):4450–63. doi: 10.1093/nar/gkac246 (PMC9071494; doi:10.1093/nar/gkac246)
Supplement: gkac246_Supplemental_Files [file gkac246_supplemental_files.zip › Supplementary Table S1 title.docx]

Supplementary Table S1

Proteins identified in 1^st^ template PIC phase, 1^st^ template multi-round transcription phase and 2^nd^ template recycling phase by LC-MS/MS
